# Seasonal transmission potential and activity peaks of the new influenza A(H1N1): a Monte Carlo likelihood analysis based on human mobility

**DOI:** 10.1186/1741-7015-7-45

**Published:** 2009-09-10

**Authors:** Duygu Balcan, Hao Hu, Bruno Goncalves, Paolo Bajardi, Chiara Poletto, Jose J Ramasco, Daniela Paolotti, Nicola Perra, Michele Tizzoni, Wouter Van den Broeck, Vittoria Colizza, Alessandro Vespignani

**Affiliations:** 1 Center for Complex Networks and Systems Research, School of Informatics and Computing, Indiana University, Bloomington, IN, USA; 2 Pervasive Technology Institute, Indiana University, Bloomington, IN, USA; 3 Department of Physics, Indiana University, Bloomington, IN, USA; 4 Computational Epidemiology Laboratory, Institute for Scientific Interchange, Turin, Italy; 5 Centre de Physique Théorique, Université d'Aix-Marseille, Marseille, France; 6 Department of Physics, University of Cagliari, Cagliari, Italy; 7 Linkalab, Cagliari, Italy; 8 Scuola di Dottorato, Politecnico di Torino, Torino Italy

## Abstract

**Background:**

On 11 June the World Health Organization officially raised the phase of pandemic alert (with regard to the new H1N1 influenza strain) to level 6. As of 19 July, 137,232 cases of the H1N1 influenza strain have been officially confirmed in 142 different countries, and the pandemic unfolding in the Southern hemisphere is now under scrutiny to gain insights about the next winter wave in the Northern hemisphere. A major challenge is pre-empted by the need to estimate the transmission potential of the virus and to assess its dependence on seasonality aspects in order to be able to use numerical models capable of projecting the spatiotemporal pattern of the pandemic.

**Methods:**

In the present work, we use a global structured metapopulation model integrating mobility and transportation data worldwide. The model considers data on 3,362 subpopulations in 220 different countries and individual mobility across them. The model generates stochastic realizations of the epidemic evolution worldwide considering 6 billion individuals, from which we can gather information such as prevalence, morbidity, number of secondary cases and number and date of imported cases for each subpopulation, all with a time resolution of 1 day. In order to estimate the transmission potential and the relevant model parameters we used the data on the chronology of the 2009 novel influenza A(H1N1). The method is based on the maximum likelihood analysis of the arrival time distribution generated by the model in 12 countries seeded by Mexico by using 1 million computationally simulated epidemics. An extended chronology including 93 countries worldwide seeded before 18 June was used to ascertain the seasonality effects.

**Results:**

We found the best estimate *R*_0 _= 1.75 (95% confidence interval (CI) 1.64 to 1.88) for the basic reproductive number. Correlation analysis allows the selection of the most probable seasonal behavior based on the observed pattern, leading to the identification of plausible scenarios for the future unfolding of the pandemic and the estimate of pandemic activity peaks in the different hemispheres. We provide estimates for the number of hospitalizations and the attack rate for the next wave as well as an extensive sensitivity analysis on the disease parameter values. We also studied the effect of systematic therapeutic use of antiviral drugs on the epidemic timeline.

**Conclusion:**

The analysis shows the potential for an early epidemic peak occurring in October/November in the Northern hemisphere, likely before large-scale vaccination campaigns could be carried out. The baseline results refer to a worst-case scenario in which additional mitigation policies are not considered. We suggest that the planning of additional mitigation policies such as systematic antiviral treatments might be the key to delay the activity peak in order to restore the effectiveness of the vaccination programs.

## Background

Estimating the transmission potential of a newly emerging virus is crucial when planning for adequate public health interventions to mitigate its spread and impact, and to forecast the expected epidemic scenarios through sophisticate computational approaches [1-4]. With the current outbreak of the new influenza A(H1N1) strain having reached pandemic proportions, the investigation of the influenza situation worldwide might provide the key to the understanding of the transmissibility observed in different regions and to the characterization of possible seasonal behavior. During the early phase of an outbreak, this task is hampered by inaccuracies and incompleteness of available information. Reporting is constrained by the difficulties in confirming large numbers of cases through specific tests and serological analysis. The cocirculation of multiple strains, the presence of asymptomatic cases that go undetected, the impossibility to monitor mild cases that do not seek health care and the possible delays in diagnosis and reporting, all worsen the situation. Early modeling approaches and statistical analysis show that the number of confirmed cases by the Mexican authorities during the early phase was underestimated by a factor ranging from one order of magnitude [5] to almost three [6]. The Centers for Disease Control (CDC) in the US estimate a 5% to 10% case detection, similar to other countries facing large outbreaks, with expected heterogeneities due to different surveillance systems. Even within the same country, the setup of enhanced monitoring led to improved notification with respect to the earlier phase of the pandemic, later relaxed as reporting requirements changed [7].

By contrast, the effort put in place by the World Health Organization (WHO) and health protection agencies worldwide is providing an unprecedented amount of data and, at last, the possibility of following in real time the pandemic chronology on the global scale. In particular, the border controls and the enhanced surveillance aimed at detecting the first cases reaching uninfected countries appear to provide more reliable and timely information with respect to the raw count of cases as local transmission occurs, and this data has already been used for early assessment of the number of cases in Mexico [5]. Moreover, data on international passenger flows from Mexico was found to display a strong correlation with confirmed H1N1 importations from Mexico [8]. Here we present an estimate of the reproduction number, *R*_0_, (that is, the average number of secondary cases produced by a primary case [9]) of the current H1N1 epidemic based on knowledge of human mobility patterns. We use the GLEaM (for GLobal Epidemic and Mobility) structured metapopulation model [10] for the worldwide evolution of the pandemic and perform a maximum likelihood analysis of the parameters against the actual chronology of newly infected countries. The method is computationally intensive as it involves a Monte Carlo generation of the distribution of arrival time of the infection in each country based on the analysis of 10^6 ^worldwide simulations of the pandemic evolution with the GLEaM model. The method shifts the burden of estimating the disease transmissibility from the incidence data, suffering notification/surveillance biases and dependent on country specific surveillance systems, to the more accurate data of the early case detection in newly affected countries. This is achieved through the modeling of human mobility patterns on the global level obtained from high quality databases. In other words, the chronology of the infection of new countries is determined by two factors. The first is the number of cases generated by the epidemic in the originating country. The second is the mobility of people from this country to the rest of the world. The mobility data are defined from the outset with great accuracy and we can therefore find the parameters of the disease spreading as those that provide the best fit for the time of infection of new countries. This method also allows for uncovering the presence of a seasonal signature in the observed pattern, not hindered or effectively caused by notification and reporting changes in each country's influenza monitoring. The obtained values for the reproduction numbers are larger than the early estimates [5], though aligned with later works [11-13]. The simulated geographic and temporal evolution of the pandemic based on these estimates shows the possibility of an early  epidemic activity peak in the Northern hemisphere as soon as mid October. While the simulations refer to a worst-case scenario, with no intervention implemented, the present findings pertain to the timing of the vaccination campaigns as planned by many countries. For this reason we also present an analysis of scenarios in which the systematic use of antiviral drug therapy is implemented with varying effectiveness, according to the national stockpiles, and study their effect on the epidemic timeline.

## Methods

**Figure 1:**
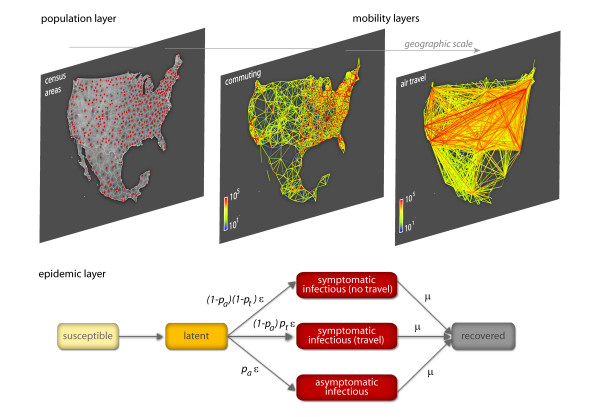
**Schematic illustration of the GLobal Epidemic and Mobility (GLEaM) model**. Top: census and mobility layers that define the subpopulations and the various types of mobility among those (commuting patterns and air travel flows). The same resolution is used worldwide. Bottom: compartmental structure in each subpopulation. A susceptible individual in contact with a symptomatic or asymptomatic infectious person contracts the infection at rate β or *r*_β_β [30,32], respectively, and enters the latent compartment where he is infected but not yet infectious. At the end of the latency period, each latent individual becomes infectious, entering the symptomatic compartments with probability 1 - *p*_a _or becoming asymptomatic with probability *p*_a _[30,32]. The symptomatic cases are further divided between those who are allowed to travel (with probability *p*_t_) and those who would stop traveling when ill (with probability 1 - *p*_t_) [30]. Infectious individuals recover permanently with rate μ. All transition processes are modeled through multinomial processes.

The GLEaM structured metapopulation model is based on a metapopulation approach [4,14-22] in which the world is divided into geographical regions defining a subpopulation network where connections among subpopulations represent the individual fluxes due to the transportation and mobility infrastructure. GLEaM integrates three different data layers [10]. The population layer is based on the high-resolution population database of the 'Gridded Population of the World' project of the SocioEconomic Data and Applications Center (SEDAC) [23] that estimates the population with a granularity given by a lattice of cells covering the whole planet at a resolution of 15 × 15 minutes of arc. The transportation mobility layer integrates air travel mobility obtained from the International Air Transport Association (IATA [24]) and Official Airline Guide (OAG [25]) databases that contain the list of worldwide airport pairs connected by direct flights and the number of available seats on any given connection [26]. The combination of the population and mobility layers allows the subdivision of the world into georeferenced census areas defined with a Voronoi tessellation procedure [27] around transportation hubs. These census areas define the subpopulations of the metapopulation modeling structure (see Figure 1). In particular, we identify 3,362 subpopulations centered around IATA airports in 220 different countries (see [10] and Additional file 1 for more details). GLEaM integrates short scale mobility between adjacent subpopulations by considering commuting patterns worldwide as obtained from the data collected and analyzed from more than 29 countries in 5 continents across the world [10]. Superimposed on these layers is the epidemic layer that defines the disease and population dynamics. The model simulates the mobility of individuals from one subpopulation to another by a stochastic procedure in which the number of passengers of each compartment traveling from a subpopulation *j *to a subpopulation *l *is an integer random variable defined by the actual data from the airline transportation database (see Additional file 1). Short range commuting between subpopulations is modeled with a time scale separation approach that defines the effective force of infections in connected subpopulations [10,28,29]. The infection dynamics takes place within each subpopulation and assumes the classic influenza-like illness compartmentalization in which each individual is classified by a discrete state such as susceptible, latent, infectious symptomatic, infectious asymptomatic or permanently recovered/removed [9,30]. The model therefore assumes that the latent period is equivalent to the incubation period and that no secondary transmissions occur during the incubation period (see Figure 1 for a detailed description of the compartmentalization). All transitions are modeled through binomial and multinomial processes to preserve the discrete and stochastic nature of the processes (see Additional file 1 for the full description). Asymptomatic individuals are considered as a fraction *p*_a _= 33% of the infectious individuals [31] generated in the model and assumed to infect with a relative infectiousness of *r*_β _= 50% [5,30,32]. Change in traveling behavior after the onset of symptoms is modeled with the probability 1 - *p*_t_, set to 50%, that individuals would stop traveling when ill [30]. The spreading rate of the disease is ultimately governed by the basic reproduction number *R*_0_. Once the disease parameters and initial conditions based on available data are defined, GLEaM allows the generation of stochastic realizations of the worldwide unfolding of the epidemic, with mobility processes entirely based on real data. The model generates *in silico *epidemics for which we can gather information such as prevalence, morbidity, number of secondary cases, number of imported cases and other quantities for each subpopulation and with a time resolution of 1 day. While global models are generally used to produce scenarios in which the basic disease parameters are defined from the outset, here we use the model to provide a maximum likelihood estimate of the transmission potential by finding the set of disease parameters that best fit the data on the arrival time of cases in different countries worldwide. It is important to stress that the model is not an agent-based model and does not include additional structure within a subpopulation, therefore it cannot provide detailed information at the level of households or workplaces. The projections for the winter season in the northern hemisphere are also assuming that there will be no mutation of the virus with respect to the spring/summer of 2009. Furthermore, while at the moment the novel H1N1 influenza is accounting for 75% of the influenza cases worldwide, the model does not consider the cocirculation of different influenza strains and cannot provide information on cocirculation data.

**Figure 2:**
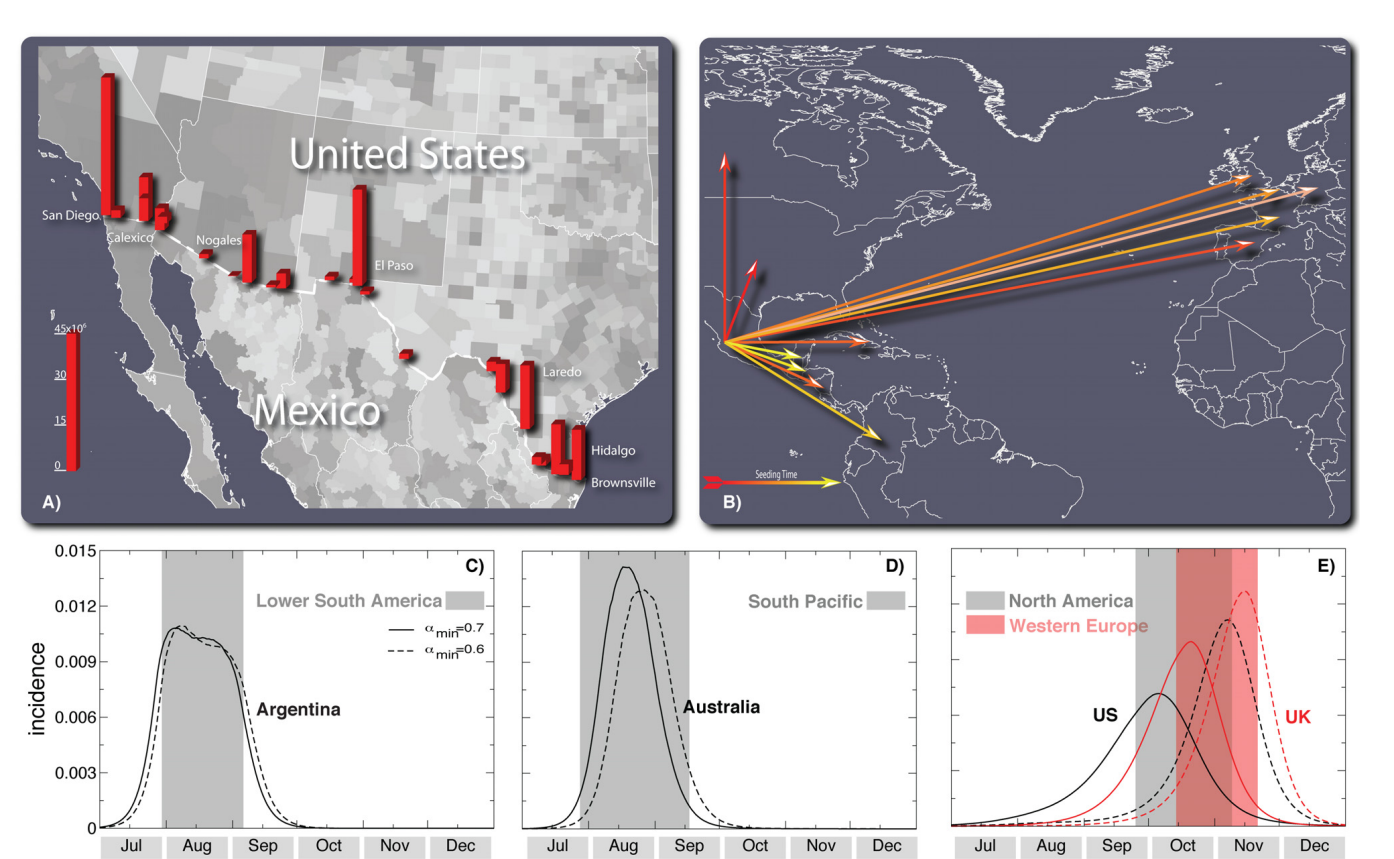
**Illustration of the model's initialization and the results for the activity peaks in three geographical areas**. **(a) **Intensity of the commuting between US and Mexico at the border of the two countries. **(b) **The 12 countries infected from Mexico used in the Monte Carlo likelihood analysis. The color scale of the arrows from red to yellow indicates the time ordering of the epidemic invasion. Panels **(c)**, **(d) **and **(e) **show the daily incidence in Lower South America, South Pacific and North America/Western Europe, respectively. The shaded area indicates the 95% confidence interval (CI) of the peak time in the corresponding geographical region. The median incidence profiles of selected countries are shown for the two values defining the best-fit seasonality scaling factor interval.

The initial conditions of the epidemic are defined by setting the onset of the outbreak near La Gloria in Mexico on 18 February 2009, as reported by official sources [33] and analogously to other works [5]. We tested different localizations of the first cases in census areas close to La Gloria without observing relevant variations with respect to the observed results. We also performed sensitivity analysis on the starting date by selecting a seeding date anticipated or delayed by 1 week with respect to the date available in official reports [33]. The arrival time of infected individuals in the countries seeded by Mexico is clearly a combination of the number of cases present in the originating country (Mexico) and the mobility network, both within Mexico and connecting Mexico with countries abroad. For this reason we integrated into our model the data on Mexico-US border commuting (see Figure 2a), which could be relevant in defining the importation of cases in the US, along with Mexican internal commuting patterns (see Figure 1) that are responsible for the diffusion of the disease from rural areas as La Gloria to transportation hubs such as Mexico City. In addition, we used a time-dependent modification of the reproductive number in Mexico as in [6] to model the control measures implemented in the country starting 24 April and ending 10 May, as those might affect the spread to other countries.

In order to ascertain the effect of seasonality on the observed pattern, we explored different seasonality schemes. The seasonality is modeled by a standard forcing that rescales the value of the basic reproductive number into a seasonally rescaled reproductive number, *R*(t), depending on time. The seasonal rescaling is time and location dependent by means of a scaling multiplicative factor generated by a sinusoidal function with a total period of 12 months oscillating in the range α_min _to α_max_, with α_max _= 1.1 days (sensitivity analysis in the range 1.0 to 1.1) and α_min _a free parameter to be estimated [17]. The rescaling function is in opposition in the Northern and Southern hemispheres (see Additional file 1 for details). No rescaling is assumed in the Tropics. The value of *R*_0 _reported in the Tables and the definition of the baseline is the reference value in the Tropics. In each subpopulation the *R*(t) relative to the corresponding geographical location and time of the year is used in the simulations.

The seasonal transmission potential of the H1N1 strain is assessed in a two-step process that first estimates the reproductive number in the Tropics region, where seasonality is assumed not to occur, by focusing on the early international seeding by Mexico, and then estimates the degree of seasonal dumping factor by examining a longer time period of international spread to allow for seasonal changes. The estimation of the reproductive number is performed through a maximum likelihood analysis of the model fitting the data of the early chronology of the H1N1 epidemic. Given a set of values of the disease parameters, we produced 2 × 10^3 ^stochastic realizations of the pandemic evolution worldwide for each *R*_0 _value. Our model explicitly takes into account the class of symptomatic and asymptomatic individuals (see Figure 1) and allows the tracking of the importation of each symptomatic individual and of the onset of symptoms of exposed individuals transitioning to the symptomatic class, as observables of the simulations. This allows us to obtain numerically with a Monte Carlo procedure the probability distribution *P*_i_(t_i_) of the importation of the first infected individual or the first occurrence of the onset of symptoms for an individual in each country i at time t_i_. Asymptomatic individuals do not contribute to the definition of t_i_. With the aim of working with conditional independent variables we restrict the likelihood analysis to 12 countries seeded from Mexico (see Figure 2b) and for which it is possible to know with good confidence the onset of symptoms and/or the arrival date of the first detected case (see Tables and data sources in Additional file 1). This allows us to define a likelihood function *L *= Π_i _*P*_i_(t_i_*), where t_i_* is the empirical arrival time from the H1N1 chronological history in each of the selected countries. This methodology assumes the prompt detection of symptomatic cases at the very beginning of the outbreak in a given country, and for this reason we have also provided a sensitivity analysis accounting for a late/missed detection of symptomatic individuals as reported in the next section. The transmission potential is estimated as the value of *R*_0 _that maximizes the likelihood function *L*, for a given set of values of the disease parameters. In Table 1 we report the reference values assumed for some of the model parameters and the range explored with the sensitivity analysis. So far there are no precise clinical estimates of the basic model parameters ε and μ defining the inverse average exposed and infectious time durations [34-36]. The generation interval *G*_t _[37,38] used in the literature is based on the early estimate of [5] and values obtained for previous pandemic and seasonal influenza [4,30-32,39,40], with most studies focusing on values ranging from 2 to 4 days [5,11-13]. We have therefore assumed a short exposed period value ε^-1 ^= 1.1 as indicated by early estimates [5] and compatible with recent studies on seasonal influenza [31,41] and performed a sensitivity analysis for values as large as ε^-1 ^= 2.5 days. The maximum likelihood procedure is performed by systematically exploring different values of the generation time aimed at providing a best estimate and confidence interval for *G*_t_, along with the estimation of the maximum likelihood value of *R*_0_.

**Table 1:** Best Estimates of the epidemiological parameters

**Parameter**	**Best Estimate**	**Interval estimate^(a)^**	**Description**
*R*_0_	1.75	1.64 to 1.88	Basic reproduction number
*G*_t_	3.6	2.2 to 5.1	Mean generation time (days)
μ^-1^	2.5	1.1 to 4.0	Mean infectious period (days)
α_min_	0.65	0.6 to 0.7	Minimal seasonality rescaling
Assumed values:			
	Assumed value at best estimate	Sensitivity analysis range	
ε^-1^	1.1	1.1 to 2.5	Mean exposed period (days)
α_max_	1.1	1.0 to 1.1	Maximum seasonality rescaling

Estimates from the Monte Carlo likelihood analyses for various values of the parameter space explored. In Additional file 1 we report the complete tables corresponding to the sensitivity analysis. (a) For *R*_0_, we report the 95% Confidence Interval. *G*_t_, μ^-1 ^intervals are defined by the range of plausible constrained values sampled in the Monte Carlo approach that satisfy a likelihood ratio test at the 5% level. The α_min _interval is the best-fit range within the minimal resolution allowed by the Monte Carlo sampling.

The major problem in the case of projections on an extended time horizon is the seasonality effect that in the long run is crucial in determining the peak of the epidemic. In order to quantify the degree of seasonality observed in the current epidemic, we estimate the minimum seasonality scaling factor α_min _of the sinusoidal forcing by extending the chronology under study and analyzing the whole data set composed of the arrival dates of the first infected case in the 93 countries affected by the outbreak as of 18 June. We studied the correlation between the simulated arrival time by country and its corresponding empirical value, by measuring the regression coefficient between the two datasets. Given the extended time frame under observation, the arrival times considered in this case are expected to provide a signature of the presence of seasonality. They included the seeding of new countries from outbreaks taking place in regions where seasonal effects might occur, as for example in the US or in the UK. For the simulated arrival times we have considered the median and 95% confidence interval (CI) emerging from the 2 × 10^3 ^stochastic runs. The regression coefficient is found to be sensitive to variations in the seasonality scaling factor, allowing discrimination of the α_min _value that best fits the real epidemic. A detailed presentation of this analysis is reported in Additional file 1. The full exploration of the phase space of epidemic parameters and seasonality scenarios reported in Additional file 1 required data from 10^6 ^simulations; the equivalent of 2 million minutes of PowerPC 970 2.5 GHz CPU time.

## Results and Discussion

Table 1 reports the results of the maximum likelihood procedure and of the correlation analysis on the arrival times for the estimation of α_min_. In the following we consider as the baseline case the set of parameters defined by the best estimates: *G*_t _= 3.6 days, μ^-1 ^= 2.5 days, *R*_0 _= 1.75.

The best estimates for *G*_t _and *R*_0 _are higher than those obtained in early findings but close to subsequent analysis on local outbreaks [11-13]. The *R*_0 _we report is the reference value for Mexico and the tropical region, whereas in each country we have to consider the *R(t) *due to the seasonality rescaling depending on the time of the year, as shown in Table 2. This might explain the lower values found in some early analysis in the US. The transmission potential emerging from our analysis is close to estimates for previous pandemics [14,42]. In Additional file 1 we provide supplementary tables for the full sensitivity analysis concerning the assumptions used in the model. Results show that larger values of the generation interval provide increasing estimates for *R*_0_. Fixing the latency period to ε^-1 ^= 1.1 days and varying the mean infectious period in the plausible range 1.1 to 4.0 days yields corresponding maximum likelihood estimates for *R*_0 _in the range 1.4 to 2.1. Variations in the latency period from ε^-1 ^= 1.1 to ε^-1 ^= 2.5 days provide corresponding best estimates for *R*_0 _in the range 1.9 to 2.3, if we assume an infectious period of 3 days. We tested variations of the compartmental model parameters *p*_a_, and *p*_t _up to 20% and explored the range *r*_β _= 20% to 80%, and sensitivity on the value of the maximum seasonality scaling factor α_max _in the range 1.0 to 1.1. The obtained estimates lie within the confidence intervals of the best estimate values.

**Table 2:** Seasonality time-dependent reproduction number in the Northern hemisphere

**Month**	***R*(t) in Northern hemisphere**
May	1.19 to 1.49
June	1.07 to 1.33
July	1.05 to 1.24
August	1.07 to 1.33
September	1.19 to 1.49

The values of *R*(t) for the Northern hemisphere correspond to the rescaling of the maximum likelihood value of *R*_0 _in Mexico and in the Tropical regions (*R*_0 _= 1.75) and the best values for the seasonality rescaling factor, 0.6 < α_min _< 0.7. The parameter α_min _indicates the minimum value of the seasonal rescaling of *R*_0 _induced by the sinusoidal forcing in the Northern hemisphere [17].

The empirical arrival time data used for the likelihood analysis are necessarily an overestimation of the actual date of the importation of cases as cases could go undetected. If we assume a shift of 7 days earlier for all arrival times available from official reports, the resulting maximum likelihood is increasing the best estimate for *R*_0 _to 1.87 (95% CI 1.73 to 2.01), as expected since earlier case importation necessitates a larger growth rate of the epidemic. The official timeline used here therefore provides, all other parameters being equal, a lower estimate of the transmission potential. We have also explored the use of a subset of the 12 countries, always generating results within the confidence interval of the best estimate.

The best estimates reported in Table 1 do not show any observable dependence on the assumption about the seasonality scenario (as reported in Additional file 1). The analysis is restricted to the first countries seeded from Mexico to preserve the conditional independence of the variables and it is natural to see the lack of any seasonal signature since these countries receive the disease from a single country, mostly found in the tropical region where no seasonal effects are expected.

In order to find the minimum seasonality scaling factor α_min _that best fits the empirical data, we performed a statistical correlation analysis of the arrival time of the infection in the 93 countries infected as of 18 June, as detailed in the Methods section and Additional file 1. By considering a larger number of countries and a longer period for the unfolding of the epidemic worldwide as seasons change, the correlation analysis for the baseline scenario provides clear statistical indications for a minimum rescaling factor in the interval 0.6 < α_min _< 0.7. In the full range of epidemic parameters explored, the correlation analysis yields values for α_min _in the range 0.4 to 0.9. This evidence for a mild seasonality rescaling is consistent with the activity observed in the months of June and July in Europe and the US where the epidemic progression has not stopped and the number of cases keeps increasing considerably (see also Table 2 for the corresponding values of *R*(t) in those regions during summer months).

This analysis allows us to provide a comparison with the epidemic activity observed so far, and most importantly an early assessment of the future unfolding of the epidemics. For each set of parameters the model generates quantities of interest such as the profile of the epidemic behavior in each subpopulation or the number of imported cases. Each simulation generates a stochastic realization of the process and the curves are the statistical aggregate of at least 2 × 10^3 ^realizations. In the following we report the median profiles and where indicated the 95% CI. For the sake of clarity data are aggregated at the level of country or geographical region. Additional file 1 reports a detailed comparison of the simulated number of cases in Australia, US, UK with the reported cases from official sources in the period May to July. Results are in good agreement with the reported temporal evolution of the epidemic and highlight a progressive decrease of the monitoring activity caused by the increasing number of cases, as expected [7]. The same information is also available for each single subpopulation defined in the model. We have therefore tested the model results in four territories of Australia. Interestingly, the model is able to recover the different timing observed in the four territories. A detailed discussion of this comparison is reported in Additional file 1.

In Figure 2c-d we report the predicted baseline case profiles for countries in the Southern hemisphere. It is possible to observe in the figure that in this case, the effect of seasonality is not discriminating between different waves, as the short time interval from the start of the outbreak to the winter season in the Southern hemisphere does not allow a large variation in the rescaling of the transmissibility during these months. Therefore we predict a first wave that occurs between August and September in phase with the seasonal influenza pattern, and independently of the seasonality parameter α_min_. The situation is expected to be different in the Northern hemisphere where different seasonality parameters might progressively shift the peak of the epidemic activity in the winter months. Figure 2e reports the predicted daily incidence profiles for the Northern hemisphere and the 95% CI for the activity peaks of the pandemic with the best-fit seasonality scenario (that is, the range 0.6 < α_min _< 0.7). Table 3 reports the same information for different continental areas. The general evidence clearly points to the occurrence of an autumn/winter wave in the Northern hemisphere strikingly earlier than expected, with peak times ranging from early October to the middle of November. The peak estimate for each geographical area is obtained from the epidemic profile summing up all subpopulations belonging to the region. The activity peak estimate for each single country can be noticeably different from the overall estimate of the corresponding geographical region as more populated areas may dominate the estimate for a given area. For instance Chile has a pandemic activity peak in the interval 1 July - 6 August, one month earlier than the average peak estimate for the Lower South America geographical area it belongs to. It is extremely important to remark that in the whole phase space of parameters explored the peak time for the epidemic activity in the Northern hemisphere lies in the range late September to late November, thus suggesting that the early seasonal peak is a genuine feature induced by the epidemic data available so far.

**Table 3:** Peak times

**Region**	**Estimated activity peak time**
North America	25 September to 9 November
Western Europe	14 October to 21 November
Lower South America	30 July to 6 September
South Pacific	28 July to 17 September

The table reports the 95% confidence interval (CI) for the pandemic activity peak time for geographical areas in the Northern and Southern hemispheres estimated for the best-fit seasonality scaling interval, 0.6 < α_min _< 0.7, and for the maximum likelihood value of *R*_0 _found for the baseline parameters, *R*_0 _= 1.75. The confidence interval is obtained from the set of numerical observations of the peak time in a given region obtained from the 2,000 stochastic runs of the model. In Additional file 1 we report the results for the full sensitivity analysis. In all cases we obtain activity peak time intervals close to those reported for the baseline scenario. Peak time estimates in this table are obtained from the epidemic profile of the entire geographical region. Single country belonging to each region could have different peak time estimates (see text).

In Table 4 we report the new number of cases at the activity peak and the epidemic size as of 15 October for a selected number of countries. As shown by the results in the table, the implementation of a massive vaccination campaign starting in October or November, with no additional mitigation implemented, would be too late with respect to the epidemic evolution, and could therefore be expected to be rather ineffective in reducing transmission. This makes a strong case for prioritized vaccination programs focusing on high-risk groups and healthcare and social infrastructure workers. In order to assess the amount of pressure on the healthcare infrastructure, in Table 5 we provide the expected number of hospitalizations at the epidemic peak according to different hospitalization rate estimates. The assessment of the hospitalization rate is very difficult as it depends on the ratio between the number of hospitalizations and the actual number of infected people. As discussed previously, the number of confirmed cases released by official agencies is always a crude underestimate of the actual number of infected people. We consider three different methods along the lines of those developed for the analysis of fatalities due to the new virus [43]. The first assumes the average value of hospitalization observed during the regular seasonal influenza season. The second is a multiplier method in which the hospitalization rate is obtained as the ratio between the WHO number of confirmed hospitalizations and the cases confirmed by the WHO multiplied by a factor 10 to 30 to account for underreporting. The third method is given by the ratio of the total number of confirmed hospitalizations and the total number of confirmed cases. This number is surely a gross overestimation of the hospitalization rate [43,44]. It has to be noted that hospitalizations are often related to existing health conditions, age and other risk factors. This implies that hospitalizations will likely not affect the population homogenously, a factor that we cannot consider in our model.

**Table 4:** Daily new number of cases and epidemic sizes in several countries

**Country**	**Peak time**	**New daily cases at the peak time (thousands)**	**New daily cases at the peak time (% of population)**	**Epidemic size at 15 October (% of population)**	
				
				**α_min _0.6**	**α_min _0.7**
United States	24 September to 9 November	2,983 to 3,302	1.06 to 1.17	4.99 to 7.38	23.76 to 29.96
Canada	4 October to 14 November	331 to 373	1.04 to 1.17	2.28 to 4.56	16.90 to 27.41
United Kingdom	9 October to 18 November	723 to 813	1.21 to 1.36	1.77 to 4.45	11.11 to 27.29
France	12 October to 21 November	725 to 792	1.26 to 1.38	1.83 to 3.87	10.86 to 26.40
Germany	11 October to 20 November	1,162 to 1,291	1.43 to 1.59	1.02 to 2.41	8.57 to 26.25
Italy	17 October to 23 November	793 to 867	1.39 to 1.52	0.93 to 2.20	6.71 to 22.13
Spain	8 October to 19 November	492 to 536	1.23 to 1.34	2.39 to 3.70	13.26 to 27.95
China	8 November to 11 December	14,077 to 16,207	1.16 to 1.34	0.65 to 5.34	1.51 to 9.49
Japan	13 October to 16 November	1,539 to 1,822	1.21 to 1.43	1.47 to 4.86	5.84 to 24.65

Peak times of the epidemic activity, daily new number of cases predicted at peak time and % of the population, and epidemic size on 15 October are shown. Intervals refer to the 95% confidence interval (CI). After 1 year from the start of the epidemic the percentage of total population infected is close to 45% with small differences of the order of 5% across different countries.

**Table 5:** Number of hospitalizations per 100,000 persons at the activity peak in several countries

	**HR based on seasonal influenza, 0.08%**	**HR based on multiplier method**		**HR based on WHO confirmed cases, 10%**
			
		**0.3%**	**1%**	
USA	2.21	8.28	27.58	275.84
Canada	2.18	8.17	27.22	272.23
UK	2.52	9.45	31.52	315.15
France	2.61	9.79	32.64	326.40
Germany	2.98	11.17	37.22	372.18
Italy	2.87	10.76	35.87	358.67
Spain	2.54	9.54	31.81	318.12
China	2.48	9.32	31.05	310.50
Japan	2.59	9.70	32.32	323.19

The estimates are obtained by considering three methods. The first assumes the average hospitalization rate (HR) observed during the seasonal influenza season. The second is a simple multiplier method in which the HR is obtained as the ratio between the World Health organization (WHO) number of confirmed hospitalizations and the cases confirmed by the WHO multiplied by a factor 10 to 30 to account for underreporting. The third method is simply the ratio of the total number of confirmed hospitalizations and the total number of confirmed cases.

**Figure 3:**
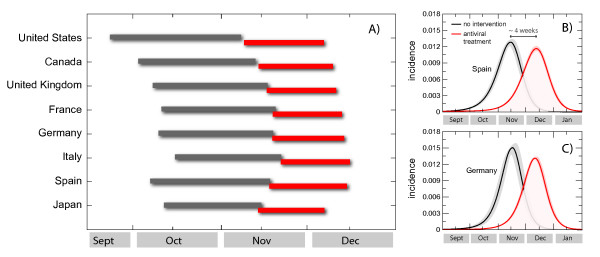
**Delay effect induced by the use of antiviral drugs for treatment with 30% case detection and drug administration**. **(a) **Peak times of the epidemic activity in the worst-case scenario (black) and in the scenario where antiviral treatment is considered (red), for a set of countries in the Northern hemisphere. The intervals correspond to the 95% confidence interval (CI) of the peak time for the two values defining the best-fit seasonality scaling factor interval. **(b, c) **Incidence profiles for Spain and Germany in the worst-case scenario (black) and in the scenario where antiviral treatment is considered (red). Results are shown for α_min _= 0.6 only, for the sake of visualization. A delay of about 4 weeks results from the implemented mitigation.

The number of hospitalized at peak times in the selected countries range between 2 and 40 per 100,000 persons, for a hospitalization rate typical of seasonal influenza and for an assumed 1% rate, respectively, yielding a quantitative indication of the potential burden that the health care systems will likely face at the peak of the epidemic activity in the next few months. It is worth noting that the present analysis considers a worst-case scenario in which no effective containment measures are introduced. This is surely not the case in that pandemic plans and mitigation strategies are considered at the national and international level. Guidelines aimed at increasing social distancing and the isolation of cases will be crucial in trying to mitigate and delay the spread in the community, thus reducing the overwhelming requests on the hospital systems. Most importantly, the mass vaccination of a large fraction of the population would strongly alter the presented picture. By contrast, any mass vaccination campaign is unlikely to start before the middle of October [45,46]. The potential for an early activity peak of the pandemic in October/November puts at risk the effectiveness of any mass vaccination program that might take place too late with respect to the pandemic wave in the Northern hemisphere. In this case it is natural to imagine the use of other mitigation strategies aimed at delaying the activity peak so that the maximum benefit can be gained with the vaccination program. As an example, we studied the implementation of systematic antiviral (AV) treatment and its effect in delaying the activity peak [19,30,32,39,47-50]. The resulting effects are clearly country specific in that each country will experience a different timing for the epidemic peak (with a local transmissibility increasing in value as we approach the winter months) and will count on antiviral stockpiles of different sizes. Here we consider the implementation of the AV treatment in all countries in the world that have drugs stockpiles available (source data from [51,52] and national agencies), until the exhaustion of their stockpiles [4]. We have modeled this mitigation policy with a conservative therapeutic successful use of drugs for 30% of symptomatic infectious individuals. The efficacy of the AV is accounted in the model by a 62% reduction in the transmissibility of the disease of an infected person under AV treatment when AV drugs are administered in a timely fashion [30,32]. We assume that the drugs are administered within 1 day of the onset of symptoms. We also consider that the AV treatment reduces the infectious period by 1 day [30,32]. In Figure 3 we show the delay obtained with the implementation of the AV treatment protocol in a subset of countries with available stockpiles. As an example, we also show the incidence profiles for the cases of Spain and Germany, where it is possible to achieve a delay of about 4 weeks with the use of 5 million and 10 million courses of AV, respectively. The results of this mitigation might be extremely valuable in providing the necessary time for the implementation of the mass vaccination program.

## Conclusion

We have defined a Monte Carlo likelihood analysis for the assessment of the seasonal transmission potential of the new A(H1N1) influenza based on the analysis of the chronology of case detection in affected countries at the early stage of the epidemic. This method allows the use of data coming from the border controls and the enhanced surveillance aimed at detecting the first cases reaching uninfected countries. This data is, in principle, more reliable than the raw count of cases provided by countries during the evolution of the epidemic. The procedure provides the necessary input to the large-scale computational model for the analysis of the unfolding of the pandemic in the future months. The analysis shows the potential for an early activity peak that strongly emphasizes the need for detailed planning for additional intervention measures, such as social distancing and antiviral drugs use, to delay the epidemic activity peak and thus increase the effectiveness of the subsequent vaccination effort.

## Competing interests

The authors declare that they have no competing interests.

## Authors' contributions

DB, HH, BG, PB and CP contributed to conceiving and designing the study, performed numerical simulations and statistical analysis, contributed to the data integration and helped to draft the manuscript. JJR contributed to conceiving and designing the study, data tracking and integration, statistical analysis and helped draft the manuscript. NP and MT contributed to data tracking and integration, statistical analysis and helped draft the manuscript. DP contributed to data integration and management and helped draft the manuscript. WVdB contributed to visualization and data management. AV and VC conceived, designed and coordinated the study, contributed to the analysis and methods development and drafted the manuscript. All authors read and approved the final manuscript.

## Pre-publication history

The pre-publication history for this paper can be accessed here:



## Supplementary Material

Additional file 1 **Additional information**. The file provides details on the model and all the statistical and sensitivity analysis carried out in the preparation of this work. The file also contains references to all data sources used in the preparation of this work. Click here for file

## References

[B1] Eubank S, Guclu H, Kumar VS, Marathe MV, Srinivasan A, Toroczkai Z, Wang N (2004). Modelling disease outbreaks in realistic urban social networks. Nature.

[B2] Ferguson NM, Cummings DA, Fraser C, Cajka JC, Cooley PC, Burke DS (2006). Strategies for mitigating an influenza pandemic. Nature.

[B3] Germann TC, Kadau K, Longini IM, Macken CA (2006). Mitigation strategies for pandemic influenza in the United States. Proc Natl Acad Sci USA.

[B4] Colizza V, Barrat A, Barthelemy M, Valleron A-J, Vespignani A (2007). Modeling the worldwide spread of pandemic influenza: baseline case and containment interventions. PloS Medicine.

[B5] Fraser C, Donnelly CA, Cauchemez S, Hanage WP, Van Kerkhove MD, Hollingsworth TD, Griffin J, Baggaley RF, Jenkins HE, Lyons EJ, Jombart T, Hinsley WR, Grassly NC, Balloux F, Ghani AC, Ferguson NM, Rambaut A, Pybus OG, Lopez-Gatell H, Alpuche-Aranda CM, Chapela IB, Zavala EP, Guevara DM, Checchi F, Garcia E, Hugonnet S, Roth C, WHO Rapid Pandemic Assessment Collaboration (2009). Pandemic potential of a strain of influenza A(H1N1): early findings. Science.

[B6] Cruz-Pacheco G, Duran L, Esteva L, Minzoni A, Lopez-Cervantes M, Panayotaros P, Ahued Ortega A, Villasenor Ruiz I (2009). Modelling of the influenza A(H1N1)v outbreak in Mexico City, April-May 2009, with control sanitary measures. Euro Surveill.

[B7] World Health Organization: Pandemic (H1N1) 2009 briefing note 3 (revised): changes in reporting requirements for pandemic (H1N1) 2009 virus infection. http://www.who.int/csr/disease/swineflu/notes/h1n1_surveillance_20090710/en/index.html.

[B8] Khan K, Arino J, Hu W, Raposo P, Sears J, Calderon F, Heidebrecht C, Macdonald M, Liauw J, Chan A, Gardam M (2009). Spread of a novel influenza A(H1N1) virus via global airline transportation. N Engl J Med.

[B9] Anderson RM, May RM (1992). Infectious diseases in humans.

[B10] Balcan D, Colizza V, Gonçalves B, Hu H, Ramasco JJ, Vespignani A (2009). Multiscale mobility networks and the large scale spreading of infectious diseases. arXiv.

[B11] Boelle PY, Bernillon P, Desenclos JC (2009). A preliminary estimation of the reproduction ratio for new influenza A(H1N1) from the outbreak in Mexico, March-April 2009. Euro Surveill.

[B12] Nishiura H, Castillo-Chavez C, Safan M, Chowell G (2009). Transmission potential of the new influenza A(H1N1) virus and its age-specificity in Japan. Euro Surveill.

[B13] Nishiura H, Wilson NM, Baker MG (2009). Estimating the reproduction number of the novel influenza A virus (H1N1) in a Southern Hemisphere setting: preliminary estimate in New Zealand. NZ Med J.

[B14] Rvachev LA, Longini IM (1985). A mathematical model for the global spread of influenza. Math Biosci.

[B15] Grais RF, Hugh Ellis J, Glass GE (2003). Assessing the impact of airline travel on the geographic spread of pandemic influenza. Eur J Epidemiol.

[B16] Hufnagel L, Brockmann D, Geisel T (2004). Forecast and control of epidemics in a globalized world. Proc Natl Acad Sci USA.

[B17] Cooper BS, Pitman RJ, Edmunds WJ, Gay N (2006). Delaying the international spread of pandemic influenza. PloS Medicine.

[B18] Epstein JM, Goedecke DM, Yu F, Morris RJ, Wagener DK, Bobashev GV (2007). Controlling pandemic flu: the value of international air travel restrictions. PLoS ONE.

[B19] Flahault A, Vergu E, Coudeville L, Grais R (2006). Strategies for containing a global influenza pandemic. Vaccine.

[B20] Viboud C, Bjornstad O, Smith DL, Simonsen L, Miller MA, Grenfell BT (2006). Synchrony, waves, and spatial hierarchies in the spread of influenza. Science.

[B21] Flahault A, Valleron A-J (1991). A method for assessing the global spread of HIV-1 infection based on air travel. Math Popul Stud.

[B22] Colizza V, Barrat A, Barthélemy M, Vespignani A (2006). The role of airline transportation network in the prediction and predictability of global epidemics. Proc Natl Acad Sci USA.

[B23] Socioeconomic Data and Applications Center (SEDAC), Columbia University. http://sedac.ciesin.columbia.edu/gpw.

[B24] International Air Transport Association. http://www.iata.org.

[B25] Official Airline Guide. http://www.oag.com/.

[B26] Barrat A, Barthélemy M, Pastor-Satorras R, Vespignani A (2004). The architecture of complex weighted networks. Proc Natl Acad Sci USA.

[B27] Okabe A, Boots B, Sugihara K, Chiu S-N (2000). Spatial Tessellations - Concepts and Applications of Voronoi Diagrams.

[B28] Keeling MJ, Rohani P (2002). Estimating spatial coupling in epidemiological systems: a mechanistic approach. Ecol Lett.

[B29] Sattenspiel L, Dietz K A structured epidemic model incorporating geographic mobility among regions. Math Biosci.

[B30] Longini IM, Halloran ME, Nizam A, Yang Y (2004). Containing pandemic influenza with antiviral agents. Am J Epidemiol.

[B31] Carrat F, Vergu E, Ferguson NM, Lemaitre M, Cauchemez S, Leach S, Valleron AJ (2008). Time lines of infection and disease in human influenza: a review of volunteer challenge studies. Am J Epidemiol.

[B32] Longini IM, Nizam A, Xu S, Ungchusak K, Hanshaoworakul W, Cummings DAT, Halloran ME (2005). Containing pandemic influenza at the source. Science.

[B33] Brote de infeccion respiratoria aguda en La Gloria, Municipio de Perote, Mexico. Secretaria de Salud, Mexico. http://portal.salud.gob.mx/contenidos/noticias/influenza/estadisticas.html.

[B34] World Health Organization (2009). WHO Weekly. Epidemiol Rec.

[B35] CDC Interim guidance for clinicians on identifying and caring for patients with swine-origin influenza A(H1N1) virus infection (2009). http://www.cdc.gov/h1n1flu/identifyingpatients.htm.

[B36] Dawood FS, Jain S, Finelli L, Shaw MW, Lindstrom S, Garten RJ, Gubareva LV, Xu X, Bridges CB, Uyeki TM, Novel Swine-Origin Influenza A (H1N1) Virus Investigation Team (2009). Emergence of a Novel Swine-origin Influenza A(H1N1) Virus in Humans. N Engl J Med.

[B37] Roberts MJ, Heesterbeek JAP (2007). Model-consistent estimation of the basic reproduction number from the incidence of an emerging infection. J Math Biol.

[B38] Wallinga J, Lipsitch M (2007). How generation intervals shape the relationship between growth rates and reproductive numbers. Proc R Soc B.

[B39] Gani R, Hughes H, Fleming D, Griffin T, Medlock J, Leach S (2005). Potential impact of antiviral drug use during influenza pandemic. Emerg Infect Dis.

[B40] Elveback LR, Fox JP, Ackerman E, Langworthy A, Boyd M, Gatewood L (1976). An influenza simulation model for immunization studies. Am J Epidemiol.

[B41] Lessler J, Reich NG, Brookmeyer R, Perl TM, Nelson KE, Cummings DA (2009). Incubation periods of acute respiratory viral infections: a systematic review. Lancet Infect Dis.

[B42] Mills CE, Robins JM, Lipsitch M (2004). Transmissibility of 1918 pandemic influenza. Nature.

[B43] Wilson N, Baker MG (2009). The emerging influenza pandemic: estimating the case fatality ratio. Euro Surveill.

[B44] Garske T, Legrand J, Donnelly CA, Ward H, Cauchemez S, Fraser C, Ferguson NM, Ghani AC (2009). Assessing the severity of the novel A/H1N1 pandemic. BMJ.

[B45] Novartis successfully demonstrates capabilities of cell-based technology for production of A(H1N1) vaccine. http://www.novartis.com/newsroom/media-releases/en/2009/1322241.shtml.

[B46] CDC: Novel H1N1 influenza vaccine. http://www.cdc.gov/h1n1flu/vaccination/public/vaccination_qa_pub.htm.

[B47] Ferguson NM, Cummings DA, Cauchemez S, Fraser C, Riley S, Meeyai A, Iamsirithaworn S, Burke DS (2005). Strategies for containing an emerging influenza pandemic in Southeast Asia. Nature.

[B48] Germann TC, Kadau K, Longini IM, Macken CA (2006). Mitigation strategies for pandemic influenza in the United States. Proc Natl Acad Sci USA.

[B49] Arinaminpathy N, McLean AR (2008). Antiviral treatment for the control of pandemic influenza: some logistical constraints. J R Soc Interface.

[B50] Wu JT, Riley S, Fraser C, Leung GM (2006). Reducing the impact of the next influenza pandemic using household-based public health interventions. PLoS Med.

[B51] Roche: update on current developments around Tamiflu. http://www.roche.com.

[B52] Singer AC, Howard BM, Johnson AC, Knowles CJ, Jackman S, Accinelli C, Caracciolo AB, Bernard I, Bird S, Boucard T, Boxall A, Brian JV, Cartmell E, Chubb C, Churchley J, Costigan S, Crane M, Dempsey MJ, Dorrington B, Ellor B, Fick J, Holmes J, Hutchinson T, Karcher F, Kelleher SL, Marsden P, Noone G, Nunn MA, Oxford J, Rachwal T (2008). Meeting report: risk assessment of Tamiflu use under pandemic conditions. Environ Health Perspect.

