# Supplementary material for: Seasonal transmission potential and activity peaks of the new influenza A(H1N1): a Monte Carlo likelihood analysis based on human mobility
Source: BMC Med. 2009 Sep 10;7:45. doi: 10.1186/1741-7015-7-45 (PMC2755471; doi:10.1186/1741-7015-7-45)
Supplement: Additional file 1 — Additional information. The file provides details on the model and all the statistical and sensitivity analysis carried out in the preparation of this work. The file also contains references to all data sources used in the preparation of this work. [file 1741-7015-7-45-S1.pdf]

Supplementary Information
Seasonal transmission potential and activity peaks of the new influenza A(H1N1): a Monte Carlo likelihood analysis based on human mobility

D Balcan, H Hu, B Gonçalves, P Bajardi, C Poletto,
JJ Ramasco, D Paolotti, N Perra, M Tizzoni, W Van den Broeck, V Colizza, A Vespignani

September 9, 2009

1 Structured metapopulation model

Here we present the detailed definition and data description of the global structured metapopulation model. The computational model is based on three data/model layers. The first layer is a data layer defining the census area and the subpopulation structure. The second one refers to human mobility model defined by the transportation and commuting networks characterizing the interactions and exchanges of individuals across subpopulations. The third layer is the epidemic dynamic model that defines the evolution of the infectious disease inside each subpopulations.

1.1 Global Population and subpopulations definition

The population dataset was obtained from the Web sites of the "Gridded Population of the World" and the "Global Urban-Rural Mapping" projects [1, 2], which are run by the Socioeconomic Data and Application Center (SEDAC) of Columbia University. The surface of the world is divided into a grid of cells that can have different resolution levels. Each of these cells has assigned an estimated population value.

Out of the possible resolutions, we have opted for cells of 15×15 minutes of arc to constitute the basis of our model. This corresponds to an area of each cell approximately equivalent to a rectangle of 25×25 kms along the Equator. The dataset comprises 823 680 cells, of which 250 206 are populated. Since the coordinates of each cell center and those of the WAN airports are known, the distance between the cells and the airports can be calculated. We have performed a Voronoi-like tessellation of the Earth surface assigning each cell to the closest airport that satisfies the following two conditions: (i) Each cell is assigned to the closest airport within the same country. And (ii), the distance between the airport and the cell cannot be longer than 200 kms. This cutoff naturally emerges from the distribution of distances between cells and closest airports, and it is introduced to avoid that in barely populated areas such as Siberia we can generate geographical census areas

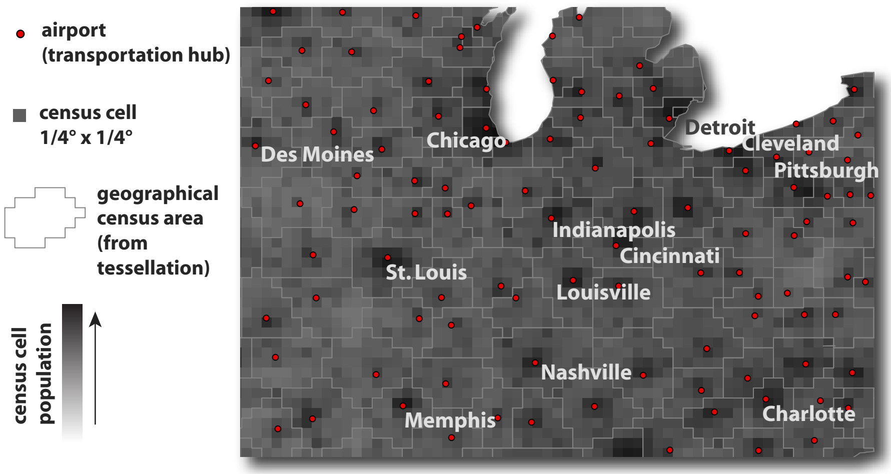

Figure 1: Population database and Voronoi tessellation around main tranportation hubs. The world surface is represented in a grid-like partition where each cell - corresponding to a population estimate - is assigned to the closest airport. Geographical census areas emerge that constitute the sub-populations of the meta-population model.

thousands of kilometer wide but with almost no population. It also corresponds to a reasonable upper cutoff for the ground traveling distance expected to be covered to reach an airport before traveling by plane.

Before proceeding with the tessellation, we need to take into account that some urban areas include more than one airport. For instance, London has up to six airport, Paris has two, and New York City has three. Our aim is to build a metapopulation model whose subpopulations correspond to the geographical census areas obtained from tessellation. Inside these geographical census areas a homogeneous mixing is assumed. The groups of airports that serve the same urban area need therefore to be aggregated since the mixing within the given urban area is expected to be high and cannot be represented in terms of separated subpopulations for each of the airports serving the same city. We have searched for groups of airports located close to each other and we manually processed the identified groups of airports to select those belonging to the same urban area. The airports of the same group are then aggregated in a single "super-hub". An example with the final result of the Voronoi tessellation procedure with cells and airports can be seen in Figure 1. The geographical census areas become thus the basic subpopulations of our metapopulation model. Their connections will determine the geographical spreading of an hypothetical epidemic. The air transportation is already integrated in the model, but a further step must be taken in order to also include ground transportation in a realistic way.

Table 1: Commuting networks in each continent. Number of countries (N_c), number of administrative units (V) and inter-links between them (E) are summarized.

Continent	N_c	V	E
Europe	17	65880	4490650
North America	2	6986	182255
Latin America	5	4301	102117
Asia	3	2732	323815
Oceania	2	746	30679
Total	29	80645	5129516

1.2 World Airport Network

The World Airport Network (WAN) is composed of 3362 commercial airports indexed by the International Air Transport Association (IATA) that are located in 220 different countries. The database contains the number of available seats per year for each direct connection between two of these airports. The coverage of the dataset is estimated to be 99% of the global commercial traffic. The WAN can be seen as a weighted graph comprising 16 846 edges whose weight, $\omega_{j\ell}$, represents the passenger flow between airports j and ℓ. The network shows a high degree of heterogeneity both in the number of destinations per airport and in the number of passengers per connection [3, 4, 5, 6].

1.3 Commuting Networks

Our commuting databases have been collected from the Offices of Statistics of 29 countries in the 5 populated continents. The full dataset comprehends more than 78 000 administrative regions and over five million commuting flow connections between them (see [7]). The definition of administrative unit and the granularity level at which the commuting data are provided enormously vary from country to country. For example, most European countries adhere to a practice that ranks administrative divisions in terms of geocoding for statistical purposes, the so called Nomenclature of Territorial Units for Statistics (NUTS). Most countries in the European Union are partitioned into three NUTS levels which usually range from states to provinces. The commuting data at this level of resolution is therefore strongly coarse-grained. In order to have a higher geographical resolution of the commuting datasets that could match the resolution scale of our geographical census areas, we looked for smaller local administrative units (LAU) in Europe. The US or Canada report commuting at the level of counties. However, even within a single country the actual extension, shape, and population of the administrative divisions are usually a consequence of historical reasons and can be strongly heterogeneous.

Such heterogeneity renders the efforts to define a universal law describing commuting flows likely to fail. The mobility behavior might indeed result different across countries simply due to the country specific partition of the population into administrative boundaries. In order to over-

come this problem, and in particular to define a data-driven short range commuting for GLEaM, we used the geographical census areas obtained from the Voronoi tessellation as the elementary units to define the centers of gravity for the process of commuting. This allows to deal with self-similar units across the world wth respect to mobility as emerged from a tessellation around main hubs of mobility and not country specific administrative boundaries.

We have therefore mapped the different levels of commuting data into the geographical census areas formed by the Voronoi-like tessellation procedure described above. The mapped commuting flows can be seen as a second transport network connecting subpopulations that are geographically close. This second network can be overlayed to the WAN in a multi-scale fashion to simulate realistic scenarios for disease spreading. The network exhibits important variability in the number of commuters on each connection as well as in the total number of commuters per geographical census area. Being the census areas relatively homogeneous and self-similar allows us to estimate a gravity law that successfully reproduce the commuting data obtained across different continents, and provide us with estimations for the possible commuting levels in the countries for which such data is not available as in ref. [7].

1.4 Epidemic dynamic model

Each geographical census area corresponds to a subpopulation in the metapopulation model, inside which we consider a Susceptible-Latent-Infectious-Recovered (SLIR) compartmental scheme, typical of influenza-like illnesses (ILIs), where each individual has a discrete disease state assigned at each moment in time. In Fig. 2, a diagram of the compartmental structure with transitions between compartments is shown. The contagion process, i.e. generation of new infections, is the only transition mechanism which is altered by short-range mobility, whereas all the other transitions between compartments are spontaneous and remain unaffected by the commuting. The rate at which a susceptible individual in subpopulation j acquires the infection, the so called force of infection λ_j, is determined by interactions with infectious persons either in the home subpopulation j or in its neighboring subpopulations on the commuting network.

Given the force of infection λ_j in subpopulation j, each person in the susceptible compartment (S_j) contracts the infection with probability $\lambda_j \Delta t$ and enters the latent compartment (L_j), where Δt is the time interval considered. Latent individuals exit the compartment with probability $\varepsilon \Delta t$, and transit to asymptomatic infectious compartment (I_j^a) with probability p_a or, with the complementary probability $1 - p_a$, become symptomatic infectious. Infectious persons with symptoms are further divided between those who can travel (I_j^t), probability p_t, and those who are travel-restricted (I_j^{nt}) with probability $1 - p_t$. All the infectious persons permanently recover with probability $\mu \Delta t$, entering the recovered compartment (R_j) in the next time step. All transitions and corresponding rates are summarized in Table 2 and in Figure 2. In each subpopulation the variation of the number of individuals in each compartment $[m]$ can be written at any given time step as

$$X_j^{[m]}(t + \Delta t) - X_j^{[m]}(t) = \Delta X_j^{[m]} + \Omega_j([m]) \quad (1)$$

where the term $\Delta X_j^{[m]}$ represents the change due to the compartment transitions induced by the disease dynamics and the transport operator $\Omega_j([m])$ represents the variations due to the traveling and mobility of individuals. The latter operator takes into account the long-range airline mobility

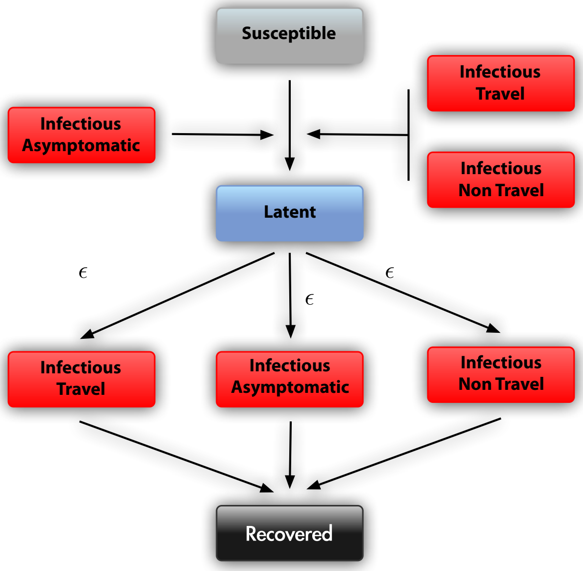

Figure 2: Compartmental structure of our epidemic model within each subpopulation.

and define the minimal time scale of integration to 1 day. The mobility due to the commuting flows is taken into account by defining effective force of infections by using a time scale separation approximations as detailed in the following sections.

The threshold parameter of the disease that determines the spreading rate of infection is called basic reproduction number (R_0), and is defined as the average number of infected cases generated by a typical infectious individual when introduced into a fully susceptible population [8]. For our compartmental model we have $R_0 = \beta\mu^{-1}[1 - p_a + r_\beta p_a]$.

1.5 Stochastic and discrete integration of the disease dynamics

In each subpopulation j, we define an operator acting on a compartment $[m]$ to account for all the transitions out of the compartment in the time interval Δt. Each element $\mathcal{D}_j([m],[n])$ of this operator is a random variable extracted from a multinomial distribution and determines the number of transitions from compartment $[m]$ to $[n]$ occurring in Δt. The change $\Delta X_j^{[m]}$ of a compartment $[m]$ in this time interval is given by a sum over all random variables $\{\mathcal{D}_j([m],[n])\}$ as follows

$$\Delta X_j^{[m]} = \sum_{[n]} \{-\mathcal{D}_j([m],[n]) + \mathcal{D}_j([n],[m])\} \quad . \tag{2}$$

As a concrete example let us consider the evolution of the latent compartment. There are three possible transitions from the compartment: transitions to the asymptomatic infectious, the symptomatic traveling and the non-traveling infectious compartments. The elements of the operator

Table 2: Transitions between compartments and their rates.

Transition	Type	Rate
$S_j \to L_j$	Contagion	λ_j
$L_j \to I_j^a$	Spontaneous	εp_a
$L_j \to I_j^t$	"	$\varepsilon(1-p_a)p_t$
$L_j \to I_j^{nt}$	"	$\varepsilon(1-p_a)(1-p_t)$
$I_j^a \to R_j$	"	μ
$I_j^t \to R_j$	"	μ
$I_j^{nt} \to R_j$	"	μ

acting on L_j are extracted from the multinomial distribution

$$Pr^{Multin}(L_j(t), p_{L_j \to I_j^a}, p_{L_j \to I_j^t}, p_{L \to I_j^{nt}}) \tag{3}$$

determined by the transition probabilities

$$\begin{aligned} p_{L_j \to I_j^a} &= \varepsilon p_a \Delta t \quad , \\ p_{L_j \to I_j^t} &= \varepsilon(1-p_a)p_t \Delta t \quad , \\ p_{L \to I_j^{nt}} &= \varepsilon(1-p_a)(1-p_t)\Delta t \quad , \end{aligned} \tag{4}$$

and by the number of individuals in the compartment $L_j(t)$ (its size). All these transitions cause a reduction in the size of the compartment. The increase in the compartment population is due to the transitions from susceptibles into latents. This is also a random number extracted from a binomial distribution

$$Pr^{Bin}(S_j(t), p_{S_j \to L_j}) \tag{5}$$

given by the chance of contagion

$$p_{S_j \to L_j} = \lambda_j \Delta t \quad , \tag{6}$$

with a number of attempts given by the number of susceptibles $S_j(t)$. After extracting these numbers from the appropriate multinomial distributions, we can calculate the change $\Delta L_j(t)$ as

$$\Delta L_j(t) = L_j(t+1) - L_j(t) = -\left[\mathcal{D}_j(L, I^a) + \mathcal{D}_j(L, I^t) + \mathcal{D}_j(L, I^{nt})\right] + \mathcal{D}_j(S, L) \ . \tag{7}$$

1.6 The integration of the transport operator

The transport operator is defined by the airline transportation data and sets the integration time scale to 1 day. The number of individuals in the compartment $[m]$ traveling from the subpopulation j to the subpopulation ℓ is an integer random variable, in that each of the X_j potential travelers

has a probability $p_{j\ell} = w_{j\ell}/N_j$ to go from j to ℓ. In each subpopulation j the numbers of individuals $\xi_{j\ell}$ traveling on each connection $j \to \ell$ at time t define a set of stochastic variables which follows the multinomial distribution

$$P(\{\xi_{j\ell}\}) = \frac{X_j^{[m]}!}{(X_j^{[m]} - \sum_\ell \xi_{j\ell})! \prod_\ell \xi_{j\ell}!} (1 - \sum_\ell p_{j\ell})^{(X_j^{[m]} - \sum_\ell \xi_{j\ell})} \prod_\ell p_{j\ell}^{\xi_{j\ell}}, \quad (8)$$

where $(1 - \sum_\ell p_{j\ell})$ is the probability of not traveling, and $(X_j^{[m]} - \sum_\ell \xi_{j\ell})$ identifies the number of non traveling individuals of the compartment $[m]$. We use standard numerical subroutines to generate random numbers of travelers following these distributions. The transport operator in each subpopulation j is therefore written as

$$\Omega_j([m]) = \sum_\ell (\xi_{\ell j}(X_\ell^{[m]}) - \xi_{j\ell}(X_j^{[m]})), \quad (9)$$

where the mean and variance of the stochastic variables are $\langle \xi_{j\ell}(X_j^{[m]}) \rangle = p_{j\ell} X_j^{[m]}$ and $\mathrm{Var}(\xi_{j\ell}(X_j^{[m]})) = p_{j\ell}(1 - p_{j\ell}) X_j^{[m]}$. It is worth remarking that on average the airline network flows are balanced so that the subpopulation N_j are constant in time, e.g. $\sum_{[m]} \Omega_j([m]) = 0$. Direct flights as well as connecting flights up to two-legs flights can be considered. Connecting flights can be generated from the original IATA data by applying a transport operator that redistribute passengers flying on a one-step and two-steps flights, based on the fraction of passengers transiting at a given airport. Values collected for this parameter and the full description of the transport operator including connecting flights can be found in Ref. [5].

1.7 Time-scale separation and the integration of the commuting flows

The Global Epidemic and Mobility (GLEaM) modeler combines the infection dynamics with long- and short-range human mobility. Each of these dynamical processes operates at a different time scale. For ILI there are two important intrinsic time scales, given by the latency period ε^{-1} and the duration of infectiousness μ^{-1}, both larger than $1\ day$. The long-range mobility given by the airline network has a time scale of the order of $1\ day$, while the commuting takes place in a time scale of approx. $\tau^{-1} \sim 1/3\ day$. The explicit implementation of the commuting in the model thus requires a time interval shorter than the minimal time of airline transportation. To overcome this problem, we use a time-scale separation technique, in which the short-time dynamics is integrated into an effective force of infection in each subpopulation.

We start by considering the temporal evolution of subpopulations linked only by commuting flows and evaluate the relaxation time to an equilibrium configuration. Consider the subpopulation j coupled by commuting to other n subpopulations. The commuting rate between the subpopulation j and each of its neighbors i will be given by σ_{ji}. The return rate of commuting individuals is set to be τ. Following the work of Sattenspiel and Dietz [9], we can divide the individuals original from the subpopulation j, N_j, between $N_{jj}(t)$ who are from j are located in j at time t and those, $N_{ji}(t)$, that are from j are located in a neighboring subpopulation i at time t. Note that by consistency

$$N_j = N_{jj}(t) + \sum_i N_{ji}(t). \quad (10)$$

The rate equations for the subpopulation size evolution are then

$$\begin{aligned} \partial_t N_{jj} = & \; -\sum_i \sigma_{ji} N_{jj}(t) + \tau \sum_i N_{ji}(t) \quad , \\ \partial_t N_{ji} = & \; \sigma_{ji} N_{jj}(t) - \tau N_{ji}(t) \quad . \end{aligned} \tag{11}$$

By using condition (10), we can derive the closed expression

$$\partial_t N_{jj} + (\tau + \sigma_j) N_{jj}(t) = N_j \tau \quad , \tag{12}$$

where σ_j denotes the total commuting rate of population j, $\sigma_j = \sum_i \sigma_{ji}$. $N_{jj}(t)$ can be expressed as

$$N_{jj}(t) = e^{-(\tau+\sigma_j)t} \left(C_{jj} + N_j \tau \int_0^t e^{(\tau+\sigma_j)s} ds \right) \quad , \tag{13}$$

where the constant C_{jj} is determined from the initial conditions, $N_{jj}(0)$. The solution for $N_{jj}(t)$ is then

$$N_{jj}(t) = \frac{N_j}{(1+\sigma_j/\tau)} + \left(N_{jj}(0) - \frac{N_j}{(1+\sigma_j/\tau)} \right) e^{-\tau(1+\sigma_j/\tau)t} \quad . \tag{14}$$

We can similarly solve the differential equation for the time evolution of $N_{ji}(t)$

$$\begin{aligned} N_{ji}(t) \; = & \; \frac{N_j \sigma_{ji}/\tau}{(1+\sigma_j/\tau)} - \frac{\sigma_{ij}}{\sigma_j} \left(N_{jj}(0) - \frac{N_j}{(1+\sigma_j/\tau)} \right) e^{-\tau(1+\sigma_j/\tau)t} \\ + & \; \left[N_{ji}(0) - \frac{N_j \sigma_{ji}/\tau}{(1+\sigma_j/\tau)} + \frac{\sigma_{ij}}{\sigma_j} \left(N_{jj}(0) - \frac{N_j}{(1+\sigma_j/\tau)} \right) \right] e^{-\tau t} \quad . \end{aligned} \tag{15}$$

The relaxation to equilibrium of N_{jj} and N_{ji} is thus controlled by the characteristic time $[\tau(1 + \sigma_j/\tau)]^{-1}$ in the exponentials. Such term is dominated by $1/\tau$ if the relation $\tau \gg \sigma_j$ holds. In our case, $\sigma_j = \sum_i \omega_{ji}/N_j$, that equals the daily total rate of commuting for the population j. Such rate is always smaller than one since only a fraction of the local population is commuting, and it is typically much smaller than $\tau \simeq 3 - 10\, day^{-1}$. Therefore the relaxation characteristic time can be safely approximated by $1/\tau$. This time is considerably smaller than the typical time for the air connections of one day and hence our approximation of considering the subpopulations $N_{jj}(t)$ and $N_{ji}(t)$ as relaxed to their equilibrium values,

$$N_{jj} = \frac{N_j}{1+\sigma_j/\tau} \quad \text{and} \quad N_{ji} = \frac{N_j \sigma_{ji}/\tau}{1+\sigma_j/\tau} \quad , \tag{16}$$

is reasonable. This approximation, originally introduced by Keeling and Rohani [10], allows us to consider each subpopulation j as having an effective number of individuals N_{ji} in contact with the individuals of the neighboring subpopulation i. In practice, this is similar to separate the commuting time scale from the other time scales in the problem (disease dynamics, traveling dynamics, etc.). While the approximation holds exactly only in the limit $\tau \to \infty$, it is good enough as long as τ^{-1} is much smaller than the typical transition rates of the disease dynamics. In the case of ILIs, the typical time scale separation between τ and the compartments transition rates is close

to one order of magnitude or even larger. The Eqs. (16) can be then generalized in the time scale separation regime to all compartments $[m]$ obtaining the general expression

$$X_{jj}^{[m]} = \frac{X_j^{[m]}}{(1+\sigma_j/\tau)} \text{ and } X_{ji}^{[m]} = \frac{X_j^{[m]}}{(1+\sigma_j/\tau)} \sigma_{ji}/\tau \,, \tag{17}$$

where $\sigma_j = \sum_{i \in \upsilon(j)} \sigma_{ji}$ denotes the total commuting rate of j. Whereas $X_{jj}^{[m]} = X_j^{[m]}$ and $X_{ji}^{[m]} = 0$ for all the other compartments which are restricted from traveling. These expressions will be used to obtain the effective force of infection taking into account the interactions generated by the commuting flows.

1.8 Effective force of infection

The force of infection λ_j that a susceptible population of a subpopulation j sees can be decomposed into two terms: λ_{jj} and λ_{ji}. The component λ_{jj} refers to the part of the force of infection whose origin is local in j. While λ_{ji} indicates the force of infection acting on susceptibles of j during their commuting travels to a neighboring subpopulation i. The effective force of infection can be estimated by summing these two terms weighted by the probabilities of finding a susceptible from j in the different locations, S_{jj}/S_j and S_{ji}/S_j, respectively. Using the time-scale separation approximation that establishes the equilibrium populations in Eq. (16), we can write

$$\lambda_j = \frac{\lambda_{jj}}{1+\sigma_j/\tau} + \sum_i \frac{\lambda_{ji}\sigma_{ji}/\tau}{1+\sigma_j/\tau} \quad . \tag{18}$$

We will focus now on the calculation of each term of the previous expression. The force of infection occurring in a subpopulation j is due to the local infectious persons staying at j or to infectious individuals from a neighboring subpopulation i visiting j and so we can write

$$\lambda_{jj} = \frac{\beta_j}{N_j^*} \left[I_{jj}^{nt} + I_{jj}^{t} + r_\beta I_{jj}^{a} + \sum_i \left(I_{ij}^{nt} + I_{ij}^{t} + r_\beta I_{ij}^{a} \right) \right] \quad , \tag{19}$$

where β_j is introduced to account for the seasonality in the infection transmission rate (if the seasonality is not considered, it is a constant), and N_j^* stands for the total effective population in the subpopulation j. By definition, $I_{jj}^{nt} = I_j^{nt}$ and $I_{ji}^{nt} = 0$ for $j \neq i$. If we use the equilibrium values of the other infectious compartments (see Eq. (16)) we obtain

$$\lambda_{jj} \;=\; \frac{\beta_j}{N_j^*} \left[I_j^{nt} + \frac{I_j^t + r_\beta I_j^a}{1+\sigma_j/\tau} + \sum_i \frac{I_i^t + r_\beta I_i^a}{1+\sigma_i/\tau} \sigma_{ij}/\tau \right] \quad . \tag{20}$$

The derivation of λ_{ji} follows from a similar argument yielding:

$$\lambda_{ji} = \frac{\beta_i}{N_i^*} \left[I_{ii}^{nt} + I_{ii}^{t} + r_\beta I_{ii}^{a} + \sum_{\ell \in \upsilon(i)} \left(I_{\ell i}^{nt} + I_{\ell i}^{t} + r_\beta I_{\ell i}^{a} \right) \right] \quad , \tag{21}$$

where $\upsilon(i)$ represents the set of neighbors of i, and therefore the terms under the sum are due to the visits of infectious individuals from the subpopulations ℓ, neighbors of i, to i. By plugging the equilibrium values of the compartment into the above expression, we obtain

$$\lambda_{ji} = \frac{\beta_i}{N_i^*}\left[I_i^{nt} + \frac{I_i^t + r_\beta I_i^a}{1+\sigma_i/\tau} + \sum_{\ell\in\upsilon(i)} \frac{I_\ell^t + r_\beta I_\ell^a}{1+\sigma_\ell/\tau}\sigma_{\ell i}/\tau\right] \quad . \tag{22}$$

Finally, in order to have an explicit form of the force of infection we need to evaluate the effective population size N_j^* in each subpopulation j, i.e., the actual number of people actually staying at the location j. The effective population is $N_j^* = N_{jj} + \sum_i N_{ij}$, that in the time-scale separation approximation reads

$$N_j^* = I_j^{nt} + \frac{N_j - I_j^{nt}}{1+\sigma_j/\tau} + \sum_i \frac{N_i - I_i^{nt}}{1+\sigma_i/\tau}\sigma_{ij}/\tau \quad . \tag{23}$$

Note that in these equations all the terms with compartments have an implicit time dependence. By inserting λ_{jj} and λ_{ji} into Eq. (18), it can be seen that the expression for the force of infection includes terms of zeroth, first and second order on the commuting ratios (i.e., σ_{ij}/τ). These three term types have a straightforward interpretation: The zeroth order terms represent the usual force of infection of the compartmental model with a single subpopulation. The first order terms account for the effective contribution generated by neighboring subpopulations with two different sources: Either susceptible individuals of subpopulation j having contacts with infectious individuals of neighboring subpopulations i, or infectious individuals of subpopulations i visiting subpopulation j. The second order terms correspond to an effective force of infection generated by the contacts of susceptible individuals of subpopulation j meeting infectious individuals of subpopulation ℓ (neighbors of i) when both are visiting subpopulation i. This last term is very small in comparison with the zeroth and first order terms, typically around two order of magnitudes smaller, and in general can be neglected.

1.9 Seasonality modeling

In order to model the seasonality effect in the northern and in the southern hemispheres, we follow the approach of Cooper *et al* [15] rescaling the basic reproduction ratio R_0 by a sinusoidal function, $s_i(t)$,

$$s_i(t) = \frac{1}{2}\left[\left(1 - \frac{\alpha_{min}}{\alpha_{max}}\right)\sin\left(\frac{2\pi}{365}(t - t_{max,i}) + \frac{\pi}{2}\right) + 1 + \frac{\alpha_{min}}{\alpha_{max}}\right] \tag{24}$$

where i refers to the hemisphere considered. In the tropical region the scaling function is identically equal to 1.0. $t_{max,i}$ is the time corresponding to the maximum of the sinusoid and hence to the maximum of the effective R_0, $R_{max} \equiv \alpha_{max}R_0$: it is fixed to Jan 15th in the northern hemisphere and six months later in the southern one. Along the year the seasonality scaling function varies from a maximum rescaling, α_{max}, to a minimum rescaling α_{min}. We have kept fixed α_{max} to 1.1 [16], corresponding to a slight increase of R_0, and tested different values of α_{min} from 0.1 to 0.9.

1.10 Control sanitary measures in Mexico

During the early stages of the epidemic, Mexican authorities implemented, under the supervision of WHO, a series of measures to increase social distance aimed at delaying the propagation of influenza. These measures consisted in school and college closure, suspension of acts involving people gathering such as concerts, masses, cinemas, etc, and the suspension for a few days of non essential economic activities. The effect of these measures has been a reduction in the number of cases reported between April 27th and May 10th and a consequent damping in the incidence curve that lasted at least until the beginning of June [17, 18].

The early stage evolution of the epidemic in Mexico is determinant for the world wide spread of the infection, therefore the authorities intervention has been taken in consideration as an ingredient of the model. We have simulated the social distances setting a low value of the basic reproductive ratio in Mexico, R_0^{Mex}, in the period between April 24th and May 10th, leaving out in this case the seasonality rescaling. In the baseline scenario we have set $R_0^{Mex} = 0.9$.

2 Likelihood analysis

We performed a Monte Carlo maximum-likelihood estimation [19, 20] of the epidemic basic reproductive number, R_0. We fit with our model the arrival dates, t_i^*, of the first infected individual in each country i. Due to the media attention, the country arrival times are the highest quality data among all the numbers regarding real epidemic cases and the least likely to be affected by under-reporting. Given our statistical model, the probability of the empirical set of arrival times $\{t_i^*\}$ conditioned to R_0, when seen as a function of R_0 itself, defines the likelihood function

$$\mathcal{L}(R_0) = \mathcal{P}(\{t_i^*\}|R_0)\,. \tag{25}$$

Maximizing this function, after fixing the values of the epidemiological and seasonality parameters $(\epsilon, \mu, \alpha_{min})$, we obtain an estimation of the basic reproductive number.

The probability distribution $\mathcal{P}(\{t_i\}|R_0)$ is embedded in the stochastic epidemic spreading process described by our model. Therefore we use a Monte Carlo methodology to sample the distribution by generating numerically $\{t_i\}$, for two thousand random realizations of the global epidemic spreading. Each stochastic run of the model starting with the same initial conditions and the same set of parameters $(\epsilon, \mu, \alpha_{min})$ yields a simulated arrival time t_i of the first symptomatic case detected in a country not yet infected. Only symptomatic cases are considered, as asymptomatic individuals would go undetected. The set of 2000 numerical observations of the arrival times t_i for each country i allows the definition of the discrete probability $P_i(t_i)$ as the fraction of runs yielding arrival time t_i. This procedure iterated for different values of R_0 allows to reconstruct the likelihood function $\mathcal{L}(R_0)$.

In order to facilitate the sampling of the distribution we have restricted the set of arrival times, considering only the 12 countries (listed in Table 5) that have been seeded by Mexico both in the real epidemic and in the simulated process (at least in the 90% of the cases). Indeed the infection arrival dates in these countries are conditional statistical independent variables, being affected only by the epidemic evolution in Mexico. Therefore in this case we can factorize $\mathcal{P}(\{t_i\})$ in the

Table 3: Maximum-likelihood estimates of the basic reproductive number, R_0, varying the seasonality factor, α_{min}, for the baseline scenario, $\epsilon^{-1} = 1.1$ and $\mu^{-1} = 2.5$.

α_{min}	R_0	[95%CI]
0.1	1.76	[1.65-1.87]
0.3	1.76	[1.65-1.87]
0.5	1.78	[1.66-1.90]
0.6	1.76	[1.64-1.88]
0.7	1.76	[1.64-1.88]
0.8	1.76	[1.65-1.87]
0.9	1.75	[1.63-1.87]
NS	1.76	[1.64-1.88]

product of the arrival time distributions for each single country

$$\mathcal{P}(\{t_i\}) = \prod_i P_i(t_i). \tag{26}$$

An example of $P_i(t_i)$ is shown in Fig. 3; it is a well peaked, smooth distribution, which allows us a good evaluation of the quantity $P_i(t_i^*)$.

In Table 3 we report the estimates of the R_0, and the corresponding confidence intervals, for the baseline scenario and different values of α_{min}. Varying the seasonality scaling we obtain always the same value of R_0 within the errors. This result is due to the fact that almost the totality of Mexico is in the tropical region and thus seasonality does not affect the epidemic dynamic within this country and consequently neither the arrival times distribution. Therefore with this procedure we are able to leave out the seasonality parameter and obtain an independent estimation of the basic reproductive number.

3 Seasonality scaling

In order to obtain a rough estimation of the seasonality scaling factor, α_{min}, we have analyzed the whole data set composed by the arrival dates of the first infected case in the 93 countries affected by the outbreak as of June 18th. This list of countries, reported in Table 6, extends the set of the 12 countries considered for the R_0 estimation in that we are interested in arrival times strongly affected by seasonality. Both in the real and in the simulated process, many of these countries are indeed seeded by the northern hemisphere where the dumping effect of seasonality had a determinant influence on the epidemic dynamic in the period from March to June spanned by our data.

We analyzed the correlation between the simulated arrival time by country and its corresponding empirical value, by measuring the regression coefficient (slope γ) between the two datasets.

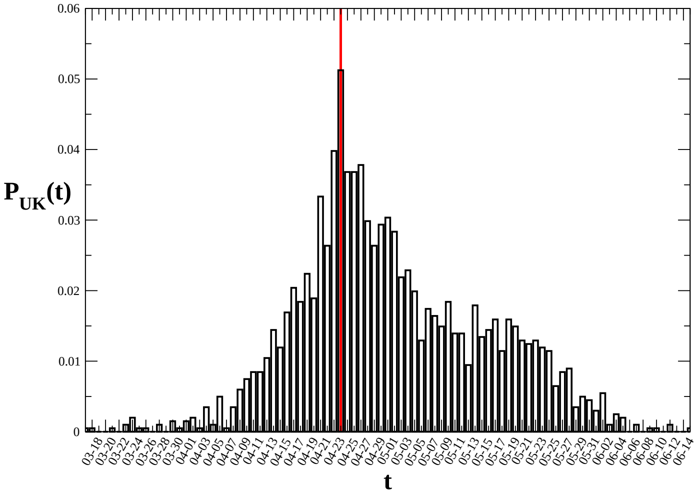

Figure 3: Distribution of the infection arrival times in the United Kingdom for the simulated spreading process corresponding to the baseline. The red bar marks the empirical data.

For the simulated arrival times we considered median and 95%CI. γ was found to be sensitive to variations in the seasonality scaling factor, allowing the discrimination of the value of α_{min} that well fit the real epidemic, as the one having the slope closer to 1. For the baseline scenario, the best correlation was found with α_{min} between 0.6 and 0.7 (the corresponding plots are reported in Fig 4). Stronger and milder seasonalities lead respectively to a slower or faster epidemic pattern in respect to epidemic data.

4 Sensitivity analysis

Sensitivity analysis has been performed on the incubation and infectiousness periods, on the application of control measures in Mexico and on the initial date of the epidemic as well as on the empirical arrival date of infection in each country. In this section we illustrate all the different scenarios we have considered. In Table 4 we report the maximum likelihood values of R_0 with the 95%CI and the estimate of the α_{min} range obtained in each case, along with the resulting activity peak times in the different regions.

All the scenarios presented in the following sections contain variations with respect to the baseline case on one or more parameters. All the parameters are set to the baseline scenario if not stated otherwise.

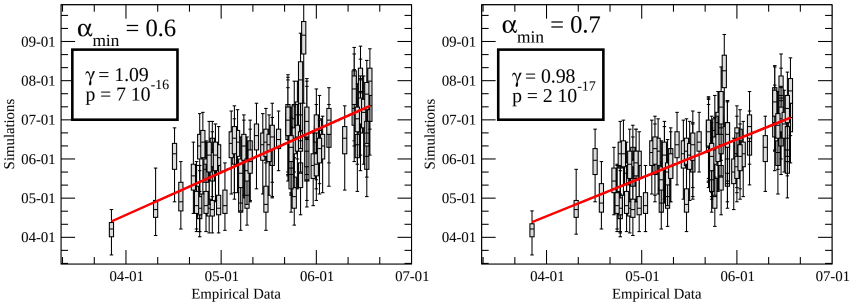

Figure 4: Simulated arrival times median and 95%CI versus the empirical ones for the two seasonality scaling factor 0.6 and 0.7.

Table 4: Results of the sensitivity analysis. Maximum likelihood value of R_0 and 95%CI, best seasonality scenarios, and predicted peak times in selected regions are shown.

**Best R_0 estimate and 95%CI**	**Range of α_{min}**	**World regions**	**Activity peak time [95%CI]**
	Shorter infectious period, $\mu^{-1} = 1.1$ days		
		North America	[Jul 25-Oct 22]
		Western Europe	[Aug 20-Nov 7]
1.42 [1.34-1.50]	0.8-0.9	Southeast Asia	[Sept 11-Nov 9]
		Upper South America	[Jul 28-Sept 15]
		Lower South America	[Jul 28-Sept 13]
		South Pacific	[Jul 22-Sept 20]
	Longer infectious period, $\mu^{-1} = 4$ days		
		North America	[Oct 19-Nov 15]
		Western Europe	[Oct 31-Nov 24]
2.11 [1.94-2.28]	0.45-0.55	Southeast Asia	[Sept 2-Oct 21]
		Upper South America	[Jul 19-Aug 24]

		Lower South America	[Jul 29-Aug 30]
		South Pacific	[Jul 28-Sept 9]
Longer latency period, $\epsilon^{-1} = 2.5$ days ($\mu^{-1} = 3$ days)			
		North America	[Oct 26-Nov 18]
		Western Europe	[Nov 7-Nov 27]
2.31 [2.14-2.48]	0.4-0.5	Southeast Asia	[Sep 7-Oct 24]
		Upper South America	[Jul 23-Aug 27]
		Lower South America	[Aug 3-Sep 2]
		South Pacific	[Aug 1-Sep 12]
Anticipated arrival time			
		North America	[Nov 13-Dec 7]
		Western Europe	[Nov 17-Dec 6]
1.87 [1.73-2.01]	0.4-0.5	Southeast Asia	[Aug 16-Oct 4]
		Upper South America	[Jul 4-Aug 4]
		Lower South America	[Jul 17-Aug 15]
		South Pacific	[Jul 15-Aug 24]
Later initial date			
		North America	[Oct 30-Nov 25]
		Western Europe	[Nov 7-Nov 27]
1.89 [1.77-2.01]	0.45-0.55	Southeast Asia	[Aug 25-Oct 12]
		Upper South America	[Jul 12-Aug 17]
		Lower South America	[Jul 22-Aug 21]
		South Pacific	[Jul 19-Sept 2]
Earlier initial date			
		North America	[Aug 3-Oct 18]
		Western Europe	[Sept 1-Nov 6]
1.65 [1.54-1.77]	0.75-0.85	Southeast Asia	[Sept 16-Nov 13]
		Upper South America	[Aug 1-Sept 17]
		Lower South America	[Aug 4-Sept 18]
		South Pacific	[Jul 28-Sept 25]

4.1 Exploration of the model parameters

We explored different values of the disease parameters in order to gain insight into the variability in the optimal value of R_0 and α_{min} and the effects that different values can have on the simulations outcome. Three scenarios have been considered. In the first two cases we fixed the latency period to $\epsilon^{-1} = 1.1$ $days$ and explored shorter and longer infectious period than the baseline value, with $\mu^{-1} = 1.1$ $days$ and $\mu^{-1} = 4$ $days$, respectively. The latter case explored a longer latency periods than the baseline, $\epsilon^{-1} = 2.5$ $days$, by fixing the infectious period at $\mu^{-1} = 3$ $days$. All results show that longer generation times (as obtained by increasing the latency period or the infectious period) yield larger values of the maximum likelihood values of R_0.

A sensitivity on the value of α_{max} in the seasonality forcing (see the subsection 1.9) in the range [1.0-1.1] provides a maximum likelihood value for R_0 that does not differ from the baseline value within its confidence interval, yielding R_0=1.77 [95%CI: 1.66-1.88]. The range of seasonality scenarios is very close to the one obtained for the baseline, with α_{min} in [0.65-0.75]. The studied variation in the winter rescaling factor therefore does not affect the baseline scenario.

We also studied variations in the relative infectiousness of asymptomatic individuals, r_β, from 20% to 80%, finding a maximum likelihood value of R_0 that is within the confidence interval of the baseline value in both cases. The obtained values are R_0=1.77 [95%CI: 1.65-1.89] for r_β=20%, and R_0=1.77 [95%CI: 1.63-1.91] for r_β=80%.

4.2 Control sanitary measures in Mexico

As part of the sensitivity analysis, we have tested different values of R_0^{Mex} (see section 1.10). Values of R^{Mex} between 0.5 and 1.2 [18] have been tested, with no relevant changes. The scenario in which no sanitary measures have been applied, i.e. $R_0^{Mex} \equiv R_0$ was also explored. While the maximum likelihood value of R_0 does not change within the error bars, yielding R_0=1.75 [95%CI: 1.64-1.86], α_{min} is decreased and is found in the range [0.45-0.55]. This result can be explained considering that most of the 93 countries taken into account for the linear regression analysis are seeded either from US or from Mexico. The detection of the seasonal effect relies on an interplay between the number of cases exported by each of these two countries. With respect to the baseline scenario, a higher effective R_0 in Mexico determines more countries seeded from this country and less from the US, which occurs with an enhanced seasonality.

4.3 Uncertainties in the chronological data

To assess the effects of the uncertainties in the chronological data, we have tested three different scenarios. We have first anticipated of one week the empirical data of the infection arrival time in each country. We consider indeed one week as the worst possible delay in the first case detection. The shift of -7 days raised the best value of R_0 to 1.87, a value that is still within the confidence interval of the maximum likelihood value of the baseline case (see Table 3 for comparison).

In order to understand the effects of variations in the beginning of the epidemic, we have simulated the scenarios in which the spreading starts one week later and one week earlier with respect to the date provided by official sources and used in the baseline scenario. February 25th and February 11th have been set as initial dates, respectively, in the two cases. As expected, the delay of one week in the pandemic initial date results in a higher value of the reproductive number with respect to the baseline scenario, with R_0 =1.89 [95%CI: 1.77-2.01]. On the other hand, the anticipation of the start of the epidemic results in lowering the value of R_0 to 1.65 [95%CI: 1.54-1.77], and moving the best values of α_{min} to $0.75 - 0.85$.

5 Chronology and data on the H1N1 worldwide spreading

In the table (5) the onset of symptoms, flight arrival and official confirmation dates are presented. We focused on 12 countries seeded by Mexico for which we could find a clear description of the

Table 5: Dates for the onset of symptoms, flight arrival and official confirmation of the first confirmed case in 12 countries seeded by Mexico are reported.

Country	Onset of symptoms	Flight arrival	Confirmed on
United States	March 28 [21]	–	April 21 [21]
Canada	April 11 [22]	April 8 [23]	April 23 [24]
El Salvador	–	April 19 [25]	May 3 [26]
United Kingdom	April 24 [27]	April 21 [28]	April 27 [24]
Spain	April 25 [29]	April 22 [30]	April 27 [24]
Cuba	–	April 25 [31]	May 13 [24]
Costa Rica	April 25 [32]	April 25 [32]	May 2 [24]
Netherlands	–	April 27 [33]	April 30 [33]
Germany	April 28 [34]	–	April 29 [24]
France	–	–	May 1 [35]
Guatemala	May 1 [36]	–	May 5 [37]
Colombia	–	–	May 3 [38]

first confirmed case. When available, official data and reports were used. When the official sources of information on a certain country were incomplete, we relied on news from the local press.

In the table (6) we show the complete timeline of confirmed cases for countries with at least one case as of July 18. The reported date corresponds to the onset of symptoms. For those countries where this information was not available, we report the flight arrival date or, when even this information was not available, we display the official confirmation date.

Table 6: Timeline of the confirmed cases as of June 18

Country	Confirmation	Ref.	Country	Confirmation	Ref.
Israel	April 26	[39]	New Zealand	April 25	[40]
Austria	April 17	[41]	Switzerland	April 29	[42]
Ireland	April 30	[43]	Denmark	April 29	[44]
Hong Kong	April 30	[45]	Italy	April 23	[46]
South Korea	April 28	[47]	Portugal	April 30	[48]
Sweden	May 6	[49]	Argentina	April 27	[50]
Poland	May 5	[51]	Japan	May 8	[52]
Panama	April 24	[53]	Brazil	May 8	[49]
Norway	May 6	[54]	Australia	May 18	[55]
Finland	May 6	[56]	Mainland China	May 9	[57]

Thailand	May 9	[58]	Belgium	May 11	[59]
Ecuador	May 10	[60]	Peru	May 10	[61]
Malaysia	May 13	[62]	Turkey	May 14	[63]
Chile	May 16	[64]	Honduras	May 11	[65]
India	May 16	[49]	Greece	May 17	[66]
Philippines	May 18	[67]	Russian Federation	May 18	[68]
Paraguay	May 20	[69]	Iceland	May 23	[70]
Kuwait	May 23	[71]	Lebanon	May 23	[72]
Romania	May 23	[73]	Bolivia	May 24	[74]
United Arab Emirates	May 24	[75]	Puerto Rico	May 24	[76]
Singapore	May 24	[77]	Bahrain	May 25	[78]
Czech Republic	May 25	[79]	Venezuela	May 25	[80]
Hungary	May 26	[81]	Vietnam	May 26	[82]
Bulgaria	May 27	[83]	Dominican Republic	May 27	[84]
Estonia	May 27	[85]	Uruguay	May 27	[86]
Slovakia	May 28	[87]	Bahamas	May 29	[88]
Cayman Islands	May 29	[89]	Cyprus	May 29	[90]
Saudi Arabia	May 29	[91]	Ukraine	May 29	[92]
Jamaica	May 31	[93]	Egypt	June 1	[94]
Luxembourg	June 1	[95]	Nicaragua	June 1	[96]
Bermuda	June 2	[97]	Trinidad and Tobago	May 30	[98]
Barbados	June 3	[99]	Dominica	June 5	[100]
French Polynesia	June 5	[101]	Morocco	June 10	[102]
Yemen	June 13	[103]	Oman	June 13	[104]
Qatar	June 13	[105]	South Africa	June 14	[106]
Suriname	June 14	[107]	Papua New Guinea	June 15	[108]
Jordan	June 15	[109]	Western Samoa	June 15	[110]
Solomon Islands	June 15	[111]	Sri Lanka	June 16	[112]
British Virgin Islands	June 17	[113]	Macau	June 17	[114]
Netherlands Antilles	June 17	[115]	Bangladesh	June 18	[116]
Lao Peoples Rep	June 18	[117]			

6 Hospitalization

The ratio between hospitalized individual and confirmed cases is probably an overestimation of the hospitalization rate (HR) because of the under-ascertainment of the real infected individuals. Furthermore, the HR shows large fluctuations from one country to another due to different monitoring systems and containment policies. As a baseline, we consider an HR=10% [118], as calculated from confirmed cases for the United States. According to ref [119] a more realistic HR can be estimated using the multiplier method. Since the number of infected individuals has been

Table 7: Weekly new cases (thousands): comparison between reported cases and simulation results.

Week	Simulated [95%CI]	Reported	[10x − 30x]
	USA		
May 10 - May 16	[0.03-11.91]	2.46	[26.60-73.80]
Jun 7 - Jun 13	[1.32-152.48]	4.64	[46.38-139.14]
Jul 5 - Jul 11	[32.16-1031.36]	3.26	[32.63-97.89]
	UK		
May 10 - May 16	[0.00-0.32]	0.05	[0.53-1.59]
Jun 7 - Jun 13	[0.02-4.35]	0.59	[5.93-17.79]
Jul 5 - Jul 11	[0.49-29.74]	2.29	[22.93-68.79]
	AUSTRALIA		
May 10 - May 16	[0.00-2.84]	0.42	[4.21-12.63]
Jun 7 - Jun 13	[0.00-151.91]	1.58	[15.77-47.31]
Jul 5 - Jul 11	[0.28-2263.80]	4.58	[45.84-137.52]

estimated to be 10-30 times larger than laboratory-confirmed cases, the HR can be calculated from real data by taking into account this scaling factor. In table 3 of the main paper values obtained for these HR are shown and compared with the one for a typical seasonal flu [120] [121].

7 Comparison of simulation results and real data

We compare the simulation results obtained for the baseline case with the reported data of a selection of countries. Table 7 shows the weekly new number of cases (expressed in thousands) for a set of three dates from early May to the end of July, for the United States, the United Kingdom, and Australia. The ranges in the simulated results correspond to the 95% CI obtained from the baseline simulations. Along with the number of reported cases, obtained from official sources [123, 124, 125, 126], we also report an interval calculated assuming a multiplier method of 10 and 30 times the confirmed cases. Results show an enhanced surveillance during the early phase of the outbreak, followed by a progressive decrease of the monitoring capacity. The larger number of cases made it indeed increasingly difficult to closely monitor the epidemic by confirming cases, and reporting requirements changed for countries that experienced an early outbreak [122].

Figure 5 reports the comparison of the epidemic evolution in 4 Territories of Australia – Western Australia, New South Wales, Queensland, and Northern Territory. The cumulative number of reported cases by Territory [126] (red circles) is compared to the corresponding predicted values, as indicated by the 95% CI (gray area). The plots show a good agreement between the empirical curves and the predicted behaviors, with the reported data being within the confidence interval of

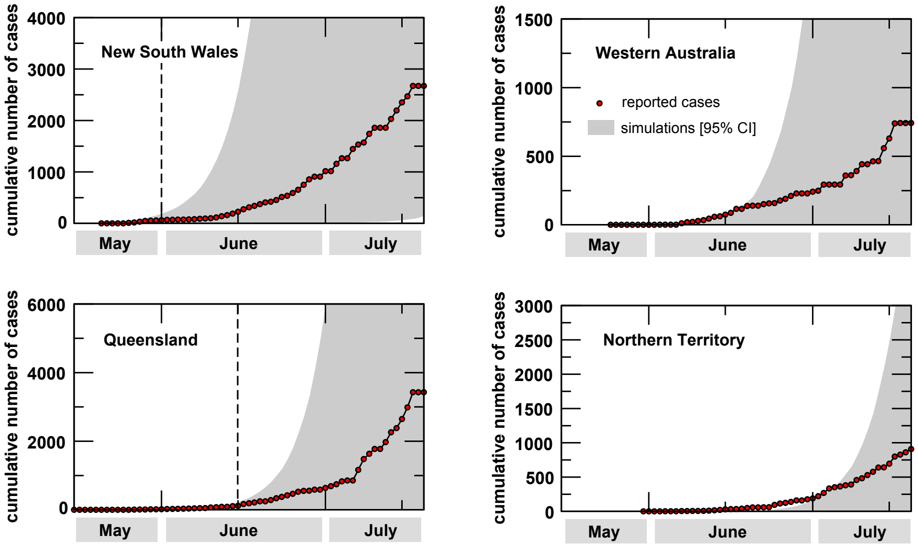

Figure 5: Comparison of reported (red circles) vs. simulated cases (95%CI indicated by the gray area) for a selection of 4 Territories of Australia. The simulations correctly predicts the different temporal evolutions observed in distinct Territories, as induced by the mobility of individuals. The vertical dashed lines indicate the delay of the start of the outbreak observed in two different Territories.

the simulations. Most importantly, the simulations are able to reproduce the heterogeneous geo-temporal pattern observed in the Australian Territories, where the outbreak started at different times. As an example, we report the time at which the first cluster of cases was detected in New South Wales and in Queensland, as indicated by the vertival dashed lines. The delay of approximately 2 weeks between the two outbreaks is correctly predicted by the simulations that take into account the mobility of individuals within the country (from actual commuting data) and with abroad.

References

[1] Center for International Earth Science Information Network (CIESIN), Columbia University; and Centro Internacional de Agricultura Tropical (CIAT). The Gridded Population of the World Version 3 (GPWv3): Population Grids. Palisades, NY: Socioeconomic Data and Applications Center (SEDAC), Columbia University. `http://sedac.ciesin.columbia.edu/gpw`

[2] Center for International Earth Science Information Network (CIESIN), Columbia University; International Food Policy Research Institute (IFPRI); The World Bank; and Centro Interna-

cional de Agricultura Tropical (CIAT). Global Rural-Urban Mapping Project (GRUMP), Alpha Version: Population Grids. Palisades, NY: Socioeconomic Data and Applications Center (SEDAC), Columbia University. `http://sedac.ciesin.columbia.edu/gpw`

[3] Barrat A, Barthélemy M, Pastor-Satorras R, Vespignani A (2004), The architecture of complex weighted networks. Proc. Natl. Acad. Sci. (USA) 101:3747—3752.

[4] Colizza V, Barrat A, Barthelemy M, Vespignani A (2006) The role of the airline transportation network in the prediction and predictability of global epidemics. Proc Natl Acad Sci U S A 103: 2015—2020.

[5] Colizza V, Barrat A, Barthelemy M, Vespignani A (2006) The modeling of global epidemics: Stochastic dynamics and predictability. Bull Math Biol 68: 1893—1921.

[6] Colizza V, Barrat A, Barthelemy M, Valleron A-J, Vespignani A (2007) Modeling the Worldwide Spread of Pandemic Influenza: Baseline Case and Containment Interventions. PLoS Med 4(1): e13. doi:10.1371/journal.pmed.0040013.

[7] D. Balcan, V. Colizza, B. Gonçalves, H. Hu, J. J. Ramasco, A. Vespignani (2009), Multiscale mobility networks and the large scale spreading of infectious diseases, arXiv:0907.3304.

[8] Anderson RM, May RM (1992), *Infectious Diseases in Humans* (Oxford Univ. Press, Oxford).

[9] Sattenspiel L, Dietz K (1995) A Structured Epidemic Model Incorporating Geographic Mobility Among Regions. Mathematical Biosciences 128: 71—91.

[10] Keeling M J, Rohani P (2002) Estimating spatial coupling in epidemiological systems: a mechanistic approach. Ecology Letters 5: 20—29.

[11] J. Wallinga, P. Teunis, and M. Kretzschmar. Using data on social contacts to estimate age-specific transmission parameters for respiratory-spread infectious agents. *Am. J. Epi.*, 164:936, 2006.

[12] International data base (idb). `http://www.census.gov/ipc/www/idb/`. Last accessed Jan 31, 2009.

[13] J. M. Heffernan R. J. Smith and L. M. Wahl. Perspectives on the basic reproductive ratio. *J. R. Soc. Interface*, 2:281, 2005.

[14] O. Diekmann, J. A. P. Heesterbeek, and J. A. J. Metz. On the definition and the computation of the basic reproduction ratio r0 in models for infectious diseases in heterogeneous populations. *J. Math. Bio.*, 28:1432, 1990.

[15] Cooper BS, Pitman RJ, Edmunds WJ, Gay NJ (2006), Delaying the International Spread of Pandemic Influenza, *PLoS Medicine* 3(6): e212.

[16] Grais RF, Ellis JH, Kress A and Glass GE (2004) Modeling the Spread of Annual Influenza Epidemics in the U.S.: The Potential Role of Air Travel, *Health Care Manag Sci* 7: 127

[17] Siatuación actual de la epidemía [Current situation of the epidemic] (July 16). Department of Health of the Mexican Government available online, in Spanish, at http://portal.salud.gob.mx.

[18] G. Cruz-Pacheco, L. Duran, L. Esteva, A.A. Minzoni, M. López-Cervantes, P. Panayotaros, A. Ahued, I. Villaseñor, *Eurosurveillance* **14**, 1 (2009).

[19] Don L. McLeish Monte Carlo simulation and finance, *Wiley*

[20] David Clayton and Micheal Hills Satistical Models in Epidemiology, *Oxford Science publications*

[21] CDC, *Briefing on Public Health Investigation of Human Cases of Swine Influenza, April 23, 2009, 3:30 p.m. ES.*, *http://www.cdc.gov/media/transcripts/2009/t090423.htm*

[22] Public Health Agency of Canada, *Cases of H1N1 Flu Virus in Canada, June 10 2009, http://www.phac-aspc.gc.ca/alert-alerte/swine-porcine/surveillance-archive/20090610-eng.php*

[23] King's-Edgehill School web site, *First cases in Canada was related to a school trip in Mexico, http://www.kes.ns.ca/flu_chronology.asp*

[24] WHO, *Chronology of Influenza A(H1N1), http://www.searo.who.int/en/Section10/Section2562_14940.htm*

[25] El Salvador Journal, *Primeros casos de gripe porcina El Salvador, May 4 2009, http://www.contrapunto.com.sv/index.php?option=com_content&view=article&id=496:primeros-casos-de-gripe-porcina-el-salvador&catid=55:categoria-ambiente&Itemid=60*

[26] Ministerio de Salud Pública y Asistencia Social, *8th official update, May 3, 2009, http://www.mspas.gob.sv/virus_gripeA_H1N1/boletines.htm*

[27] ABC news, *Swine flu cases confirmed in Scotland, April 28, http://www.abc.net.au/news/stories/2009/04/28/2554208.htm*

[28] The Scottish Government, *Scottish Government News Release, April 26, http://www.scotland.gov.uk/News/Releases/2009/04/26164218*

[29] Ministerio De Sanidad y Political Social, *Official Update, April 27 2009, http://www.msc.es/servCiudadanos/alertas/comunicadosNuevaGripe.jsp?time=1238536800000*

[30] The Guardian, *Spain confirms first swine flu case in Europe, April 27 2009, http://www.guardian.co.uk/world/2009/apr/27/swine-flu-spain-europe*

[31] Usa Today, *Cuba confirms its 1st swine flu case, May 12 2009, http://www.usatoday.com/news/world/2009-05-11-cuba_N.htm*

[32] Global Post, *Costa Rica reports first swine flu case, April 28 2009,* http://www.globalpost.com/notebook/costa-rica/090428/costa-rica-reports-first-swine-flu-case

[33] Ministry of Health Welfare and Sport (Netherlands), *First victim Mexican flu, April 30 2009,* http://www.minvws.nl/en/nieuwsberichten/pg/2009/first-victim-mexican-flu.asp

[34] Robert Koch Institut, *Neue Influenza A/H1N1 in Deutschland Bewertung des bisherigen Geschehens,* http://www.rki.de/cln_091/nn_200120/DE/Content/Infekt/EpidBull/Archiv/2009/25/Art__01.html

[35] Ministère de la Santé et des Sports, *Official Update, May 1,* http://www.sante-sports.gouv.fr/actualite-presse/presse-sante/communiques/bulletin-quotidien-nouvelle-grippe-h1n1-dite-porcine.html

[36] El Periodico Guatemala, *Ministro de Salud confirma primer caso de AH1N1 en Guatemala, May 5 2009,* http://www.elperiodico.com.gt/es/20090505/pais/99779/

[37] Ministerio de Salud Pública de Guatemala, *Official Update, May 5 2009,* http://portal.mspas.gob.gt/index.php?ID=6128&action=display&ID_BOLETIN=61

[38] Ministerio de la Protecciìon Social República de Colombia, *Official Update, May 3 2009,* http://www.minproteccionsocial.gov.co/VBeContent/NewsDetail.asp?ID=18582&IDCompany=3

[39] Thaindian News, *Israel confirms first case of swine flu , April 28, 2009,* http://www.thaindian.com/newsportal/health1/israel-confirms-first-case-of-swine-flu_100185671.html

[40] The New Zealand Herald, *NZ students in swine flu scare (Cases confermed on April 28 by the Ministry of Health), April 26, 2009,* http://www.nzherald.co.nz/nz/news/article.cfm?c_id=1&objectid=10568751

[41] China Daily, *First suspected case of swine flu in Austria (confirmed on April 29), April 28, 2009* http://www.chinadaily.com.cn/world/2009-04/28/content_7726033.htm

[42] SwissInfo.ch, *Swine flu reaches Switzerland, April 30, 2009* http://www.swissinfo.ch/eng/front/Swine_flu_reaches_Switzerland.html?siteSect=105&sid=10638163&cKey=1241072742000&ty=st

[43] Times of India, *Ireland confirms first swine flu case, May 3, 2009* http://timesofindia.indiatimes.com/World/Europe/Ireland-confirms-first-swine-flu-case/articleshow/4478077.cms

[44] Bt.dk News, *Dansk kvinde smittet med H1N1-influenza efter New York-rejse (Health confirms that a Dane infected with H1N1 after a trip to the United States), May 1, 2009* http://www.bt.dk/nyheder/dansk-kvinde-smittet-med-h1n1-influenza-efter-new-york-rejse

[45] ABC News, *Hong Kong, Denmark confirm first swine flu cases, May 2, 2009* http://www.abc.net.au/news/stories/2009/05/02/2558882.htm?section=world

[46] AFP News, *First swine flu case confirmed in Italy, May 2, 2009* http://www.google.com/hostednews/afp/article/ALeqM5ih698-EtG41Byo_RrqPMvQ2qiW4g

[47] The Jakarta Post, *South Korea has first confirmed swine flu case , May 2, 2009* http://www.thejakartapost.com/news/2009/05/02/south-korea-has-first-confirmed-swine-flu-case.html

[48] Correio do Minho, *Gripe H1N1: Primeiro caso confirmado em Portugal , May 4, 2009* http://www.correiodominho.com/noticias.php?id=6322

[49] WHO, *Chronology of Influenza,* http://www.searo.who.int/en/Section10/Section2562_14943.htm#may4

[50] Reuters, *Argentina confirms first H1N1 flu case,* http://www.reuters.com/article/GCA-SwineFlu/idUSTRE5468RC20090507

[51] Poland.pl, *First case of swine flu in Poland, May 7 2009* http://www.poland.pl/news/article,First_case_of_swine_flu_in_Poland,id,376309.htm

[52] Fox News, *Japan, Australia Confirm First Cases of Swine Flu, May 9 2009* http://www.foxnews.com/story/0,2933,519597,00.html

[53] Thaindian News, *Panama reports first swine flu case, May 9 2009* http://www.thaindian.com/newsportal/health1/panama-reports-first-swine-flu-case_100190134.html

[54] Dagsavisen, *Vurderer a lempe pa reiserad for Mexico, May 10 2009* http://www.dagsavisen.no/innenriks/article414972.ece

[55] TheAge.com.au, *Swine flu: three brothers test positive, May 20 2009* http://www.theage.com.au/national/swine-flu-three-brothers-test-positive-20090520-bf2q.html

[56] YLE.fi, *Finland Confirms First Two Swine Flu Cases, May 12 2009* http://yle.fi/uutiset/news/2009/05/finland_confirms_first_two_swine_flu_cases_733734.html

[57] EEO.com.cn, *Mainland China's First Case of Swine Flu Confirmed , May 11 2009* http://www.eeo.com.cn/ens/biz_commentary/2009/05/11/137302.shtml

[58] Mysinchew, *Thailand reports first confirmed case of swine flu , May 12 2009* http://www.mysinchew.com/node/24372?tid=14

[59] Belgium Health Dept., *Official Updated , May 13 2009* http://www.influenza.be/nl/_documents/2009-05-13_Persbericht23_stand_van_zaken_NL.pdf

[60] Ministerio de Salud Pública, *Official Updated, May 15 2009* http://www.msp.gov.ec/index.php?option=com_content&task=view&id=601&Itemid=84

[61] Ministerio de Salud Publica, *Official Updated, May 15 2009* http://www.minsa.gob.pe/portada/prensa.htm

[62] The Star, *Malaysia confirms first case of A(H1N1) flu (Update 2), May 15 2009* http://thestar.com.my/news/story.asp?file=/2009/5/15/nation/20090515141134

[63] Turkishny, *First case of swine flu confirmed in Turkey (health minister), May 16 2009* http://www.turkishny.com/en/english-news/8098-first-case-of-swine-flu-confirmed-in-turkey-health-minister.html

[64] Gobierno de Chile Ministerio de Salud, *Official Report, May 17 2009* http://www.pandemia.cl/pagnew/prensa/REPORTE_17_05_09.pdf

[65] El Nacional, *Honduras anunciò su primer caso de gripe A, May 21 2009,* http://el-nacional.com/www/site/p_contenido.php?q=nodo/82382/Internacional/Honduras-anunci%C3%B3-su-primer-caso-de-gripe-A

[66] Sofia Echo, *Greece confirms first swine flu case, May 19 2009,* http://sofiaecho.com/2009/05/19/721446_greece-confirms-first-swine-flu-case

[67] Global Nation, *First swine flu virus case in RP, May 22 2009,* http://globalnation.inquirer.net/cebudailynews/news/view/20090522-206490/First-swine-flu-virus-case-in-RP

[68] Ekaterinburg, *First case of swine flu confirmed in Russia, May 25 2009,* http://www.ekaterinburg.com/news/print/news_id-303076.html

[69] Momento 24, *Paraguay: primer caso confirmado de gripe A, May 20 2009,* http://momento24.com/2009/05/20/paraguay-primer-caso-confirmado-de-gripe-a/

[70] Bnlz, *Iceland confirms 1st swine flu case, May 23 2009,* http://www.blnz.com/news/2009/05/23/Iceland_confirms_swine_case_3689.html

[71] Al Arabiya, *Kuwait reports swine flu cases in US soldiers, May 23 2009,* http://www.alarabiya.net/articles/2009/05/23/73642.html

[72] AFP News, *Lebanon confirms three swine flu cases, May 30 2009,* http://www.google.com/hostednews/afp/article/ALeqM5iydVpJBp6UhDs2ppxJx5Ddjakd2Q

[73] The Star, *Romania has first confirmed cases of swine flu, June 1 2009,* http://thestar.com.my/news/story.asp?file=/2009/6/1/apworld/20090601154306&sec=apworld

[74] Ministerio de Salud Y Deportes, *Official Update, June 2 2009,* http://www.sns.gov.bo/documentos/epidemiologia/2009_6_3_1.pdf

[75] Anadubawi, *First case of swine flu in UAE, May 24 2009,* http://www.anadubawi.com/dubai_discussions/hanging_out_place/first_case_swine_flu_uae_dubai

[76] Zimbio, *Puerto Rico reports 1st swine flu case, May 26 2009*, http://www.zimbio.com/Judy+Trunnell/articles/22/Puerto+Rico+reports+1st+swine+flu+case

[77] Groud Report, *Singapore confirms first case of swine flu, May 26 2009*, http://www.groundreport.com/World/Singapore-confirms-first-case-of-swine-flu

[78] Arabian Business, *First Bahrain swine flu case confirmed, May 26 2009*, http://www.arabianbusiness.com/556747-first-bahrain-swine-flu-case-confirmed---tv

[79] Aktualne, *Czech Republic confirms first case of swine flu, May 25 2009*, http://aktualne.centrum.cz/czechnews/clanek.phtml?id=638252

[80] Ministerio del Poder Popular para la Salud, *Official Update, May 28 2009*, http://www.mpps.gob.ve/ms/modules.php?name=News&file=article&sid=2143

[81] World News, *Hungary reports first case of swine flu, May 29 2009*, http://article.wn.com/view/2009/05/29/Hungary_reports_first_case_of_swine_flu/

[82] The Irrawaddy, *Vietnam Reports First Swine Flu Case, June 1 2009*, http://www.irrawaddy.org/article.php?art_id=15795

[83] VisitBulgaria.info, *Bulgaria confirms its first case of swine flu, June 1 2009*, http://visitbulgaria.info/10147-bulgaria-confirms-its-first-case-swine-flu

[84] World News, *Dominican Republic confirms first swine flu cases, May 27 2009*, http://article.wn.com/view/2009/05/27/Dominican_Republic_confirms_first_swine_flu_cases/

[85] VisitBulgaria.info, *First swine flu case confirmed in Estonia, May 30 2009*, http://visitbulgaria.info/10104-first-swine-flu-case-confirmed-estonia

[86] Topix, *Swine flu: Uruguay confirms first cases, May 27 2009*, http://www.topix.com/world/uruguay/2009/05/swine-flu-uruguay-confirms-first-cases

[87] Top News, *Slovakia reports first swine flu case, May 28 2009*, http://www.topnews.in/slovakia-reports-first-swine-flu-case-2172271

[88] Blnz, *Bahamas, Bermuda report 1st swine flu cases, June 6 2009*, http://www.blnz.com/news/2009/06/02/Bahamas_Bermuda_report_swine_cases_2839.html

[89] Living Cayman, *First Swine FLU Case in Cayman*, http://www.livingcayman.com/first-swine-flu-case-in-cayman/

[90] Times of India, *Cyprus confirms first case of swine flu, May 30 2009*, http://timesofindia.indiatimes.com/World/Rest-of-World/Cyprus-confirms-first-case-of-swine-flu/articleshow/4598634.cms

[91] Arabian Business, *Saudi confirms first swine flu case, June 3 2009*, http://www.arabianbusiness.com/557651-saudi-confirms-first-swine-flu-case

[92] M&C, *Ukraine confirms its first case of swine flu, June 2 2009*, http://www.monstersandcritics.com/news/health/news/article_1480910.php/Ukraine_confirms_its_first_case_of_swine_flu_

[93] World News, *Jamaica reports 1st confirmed swine flu cases, June 2 2009*, http://article.wn.com/view/2009/05/31/Jamaica_reports_1st_confirmed_swine_flu_cases/

[94] Voa News, *Egypt Reports 1st Swine Flu Case, June 2 2009*, http://www.voanews.com/english/archive/2009-06/2009-06-02-voa55.cfm?CFID=258242880&CFTOKEN=53711156&jsessionid=8830810cfd80a9177bb79467251f2d03984f

[95] Reuters, *Luxembourg confirms first H1N1 flu case, June 2 2009*, http://www.alertnet.org/thenews/newsdesk/L2466685.htm

[96] FluInfection.net, *Nicaragua registers first case of A/H1N1 flu, June 2 2009*, http://www.fluinfection.net/nicaragua-registers-first-case-of-ah1n1-flu

[97] Blnz, *Bahamas, Bermuda report 1st swine flu cases, June 2 2009*, http://www.blnz.com/news/2009/06/02/Bahamas_Bermuda_report_swine_cases_2839.html

[98] Trinidad Express, *Call for Flight 1647 passengers to contact 'H1N1' hotline, June 7 2009*, http://www.trinidadexpress.com/index.pl/article_news?id=161487721

[99] The Star News, *Barbados confirms first swine flu case, June 4 2009*, http://thestar.com.my/news/story.asp?file=/2009/6/4/apworld/20090604103316&sec=apworld

[100] eTaiwan News, *Dominica confirms 1st case of swine flu, June 8 2009*, http://www.etaiwannews.com/etn/news_content.php?id=971121&lang=eng_news

[101] Australian Network News, *French Polynesia confirms first swine flu case, June 5 2009*, http://australianetworknews.com/stories/200906/2590738.htm?desktop

[102] The Times, *Morocco reports first swine case, June 12 2009*, http://www.thetimes.co.za/News/Article.aspx?id=1016885

[103] M&C, *Yemen reports first confirmed swine flu case, June 16 2009*, http://www.monstersandcritics.com/news/health/news/article_1483956.php/Yemen_reports_first_confirmed_swine_flu_case_

[104] The National, *Oman reports first swine flu cases, June 17 2009*, http://www.thenational.ae/article/20090617/FOREIGN/706179922

[105] Middle East Online, *Qatar reports first swine flu cases, June 16 2009*, http://www.middle-east-online.com/english/yemen/?id=32781

[106] News Today, *South Africa confirms first swine flu case, June 18 2009*, http://www.newstoday.co.za/cgi-bin/newstoday/show.pl?1245328145

[107] Caribbean Net News, *Suriname confirms first cases of swine flu, June 16 2009,* `http://www.caribbeannetnews.com/news-17121--36-36--.html`

[108] Nine MSN, *One Papua New Guinea case of swine flu confirmed, June 19 2009,* `http://news.ninemsn.com.au/health/827514/one-png-case-of-swine-flu-confirmed`

[109] Reuters, *Jordan, Qatar, Yemen identify first H1N1 flu cases, June 16 2009,* `http://www.reuters.com/article/GCA-SwineFlu/idUSTRE55F3LJ20090616`

[110] Talamua, *Samoa's first positive case of swine flu, June 16 2009,* `http://www.talamua.com/index.php?option=com_content&view=article&id=466:samoas-first-positive-case-of-swine-flu&catid=1:headlines&Itemid=33`

[111] Australian Network News, *Solomon Islands may lack capacity to deal with H1N1 outbreak says official, June 15 2009,* `http://australianetworknews.com/stories/200906/2598896.htm?desktop`

[112] Sri Lanka Today, *First Swine Flu case, June 17 2009,* `http://srilankatoday.com/content/view/2968/52/`

[113] Virgin Island Daily News, *First case of swine flu reported in U.S. Virgin Islands, June 17 2009,* `http://www.virginislandsdailynews.com/index.pl/article_home?id=17636878`

[114] The Standard, *Macau hit by first swine flu case, June 18 2009,* `http://www.thestandard.com.hk/breaking_news_detail.asp?id=15172`

[115] International SOS, *Netherlands Antilles: first case, in St. Maarten, June 17 2009,* `http://www.internationalsos.com/pandemicpreparedness/CountryLevel.aspx?languageID=ENG&countryID=210&catID=27`

[116] Thaindian News, *First swine flu case detected in Bangladesh, June 19 2009,* `http://www.thaindian.com/newsportal/health1/first-swine-flu-case-detected-in-bangladesh_100206976.html`

[117] Asia One, *Laos reports first swine flu case, June 18 2009,* `http://www.asiaone.com/Health/News/Story/A1Story20090618-149350.html`

[118] CDC, *2008-2009 influenza epidemiology; seasonal and novel influenza A(H1N1)*

[119] N. Wilson, M.G. Baker, *The emerging influenza pandemic: estimating the case fatality ratio*, Eurosurveillance, Vol. 14, Issue 26, 2 July 2009

[120] N.A.M. Molinari et al., *doi:10.1016/j.vaccine.2007.03.046*

[121] CDC, *Prevention and control of influenza: recommendations of the Advisory Committee on Immunizations Practices*, MMWR 2006;55(RR10):1-42

[122] WHO, *Pandemic (H1N1) 2009 briefing note 3 (revised): Changes in reporting requirements for pandemic (H1N1) 2009 virus infection*, `http://www.who.int/csr/disease/swineflu/notes/h1n1_surveillance_20090710/en/index.html`.

[123] CDC, *H1N1 Flu Daily Updates*, `http://cdc.gov/h1n1flu/updates`

[124] Health Protection Agency, *National Press Releases*, `http://www.hpa.org.uk`

[125] ECDC, *Pandemic (H1N1) 2009*, `http://www.ecdc.europa.eu/en/healthtopics/Pages/Influenza_A\%28H1N1\%29_Outbreak.aspx`

[126] Australian Government, Department of Health and Ageing, *Update bulletins for Pandemic (H1N1) 2009*, `http://www.healthemergency.gov.au/internet/healthemergency/publishing.nsf/Content/updates`
